# Transcriptome Wide Annotation of Eukaryotic RNase III Reactivity and Degradation Signals

**DOI:** 10.1371/journal.pgen.1005000

**Published:** 2015-02-13

**Authors:** Jules Gagnon, Mathieu Lavoie, Mathieu Catala, Francis Malenfant, Sherif Abou Elela

**Affiliations:** Université de Sherbrooke Centre of Excellence in RNA Biology, Département de microbiologie et d’infectiologie, Faculté de médecine et des sciences de la santé, Université de Sherbrooke, Sherbrooke, Québec, Canada; Cornell, UNITED STATES

## Abstract

Detection and validation of the RNA degradation signals controlling transcriptome stability are essential steps for understanding how cells regulate gene expression. Here we present complete genomic and biochemical annotations of the signals required for RNA degradation by the dsRNA specific ribonuclease III (Rnt1p) and examine its impact on transcriptome expression. Rnt1p cleavage signals are randomly distributed in the yeast genome, and encompass a wide variety of sequences, indicating that transcriptome stability is not determined by the recurrence of a fixed cleavage motif. Instead, RNA reactivity is defined by the sequence and structural context in which the cleavage sites are located. Reactive signals are often associated with transiently expressed genes, and their impact on RNA expression is linked to growth conditions. Together, the data suggest that Rnt1p reactivity is triggered by malleable RNA degradation signals that permit dynamic response to changes in growth conditions.

## Author Summary

RNA degradation is essential for gene regulation. The amount and timing of protein synthesis is determined, at least in part, by messenger RNA stability. Although RNA stability is determined by specific structural and sequence motif, the distribution of the degradation signals in eukaryotic genomes remains unclear. In this study, we describe the genomic distribution of the RNA degradation signals required for selective nuclear degradation in yeast. The results indicate that most RNAs in the yeast transcriptome are predisposed for degradation, but only few are catalytically active. The catalytic reactivity of messenger RNAs were mostly determined by the overall structural context of the degradation signals. Strikingly, most active RNA degradation signals are found in genes associated with respiration and fermentation. Overall, the findings reported here demonstrate how certain RNA are selected for cleavage and illustrated the importance of this selective RNA degradation for fine tuning gene expression in response to changes in growth condition.

## Introduction

RNA stability is a critical determinant of gene expression required for the adjustment of RNA abundance in response to changes in growth conditions [1]. Alterations of mRNA stability are associated with many gene expression programs like T cell activation [2], response to osmotic shock [3] and change in carbon source [4]. In addition, selective RNA degradation was shown to play a central role in both cellular and organismal development underlining the importance of this process to the gene expression program [5]. However, despite these profound effects on cell function and growth, the mechanisms by which specific transcripts are selected for degradation remain unclear. RNAs with similar degradation or processing signals often display distinct decay profiles and respond to different cellular cues [6]. Attempts to define the features required for selective RNA degradation are seriously hindered by the limited understanding of the ribonucleases involved in those processes.

In general, RNA turnover and quality control are achieved by exoribonucleases which are mostly controlled by the accessibility of the substrate’s 5’ and 3’ ends [7]. On the other hand, conditional degradation of RNA molecules is often triggered by endoribonucleases that accurately identify specific sequences or structures at a particular time or growth condition [8]. The most studied of these selective endoribonucleases are members of the dsRNA specific ribonuclease III (RNase III) family, which was first discovered in bacteria [9]. These ubiquitous enzymes are defined by their homology to structural elements, which include a nuclease domain (RIIID) that exhibits a conserved divalent metal binding motif, and a double-stranded RNA binding domain (dsRBD) [10]. In bacteria, RNase III regulates the expression of many conditionally expressed genes like those implicated in metal transport [11] and fermentative growth [12]. Similarly, baker’s yeast RNase III (Rnt1p) directly cleaves the mRNA of genes implicated in glucose sensing [13,14], cell cycle and cell wall stress response [15]. In metazoans, the RNase III enzymes Drosha and Dicer are required for the processing of the short non-coding RNA needed for sequence specific RNA degradation [16,17].

The sequence and structural features of natural substrates are hard to identify for most RNase IIIs. Studies of *E*. *coli* RNase III suggest that substrate selection is influenced by antideterminant nucleotides (nucleotides that deter cleavage) [18]. On the other hand, eukaryotic RNase IIIs possess more specific mechanisms of substrate selectivity. For example, human Dicer recognizes terminal loops and RNA ends and its substrate specificity is modified *in vivo* by protein factors like TRBP and PACT [19,20]. Similarly, substrate recognition by Drosha requires a combination of RNA structure and chaperon proteins [8,21]. The most selective enzyme among the members of the RNase III family is found in yeast *Saccharomyces cerevisiae*. Rnt1p prefers short stem loop structures, capped with either NGNN tetraloop (G2-loop) [10,22,23] or AAGU (A1-loop) structures to long RNA duplexes [22]. Deletion or mutation of these loops block cleavage and reduce binding under physiological conditions [10,24]. This apparently strict substrate specificity suggests that Rnt1p has fewer and more homogeneous targets than other RNase III. However, our knowledge of Rnt1p substrates was deduced from a relatively small number of related substrates (e.g. snoRNAs) [25] that may not reflect the entire spectrum of the enzyme reactivity. Indeed, the broad impact of *RNT1* deletion on yeast phenotypic behavior and transcriptome suggests that Rnt1p reactivity is not restricted to non-coding RNA processing [13,26]. This is consistent with the fact that Rnt1p is the only homologue of RNase III proteins in *S*. *cerevisiae*.

In this study, we used a combination of genome-wide analysis techniques to outline the overall contribution of Rnt1p to the regulation of gene expression in *S*. *cerevisiae* and define the nature of its cleavage signals. Direct cleavage assay of the entire transcriptome permitted unbiased characterization of Rnt1p reactivity *in vitro* and defined the predisposition of all transcripts to selective RNA degradation. The results indicate that although Rnt1p cleavage signals are randomly distributed across the yeast genome, only 10% of the genes are upregulated *in vivo* in the absence of *RNT1* and 5% are directly cleaved by the recombinant enzyme *in vitro*. Many of the newly identified cleavage sites were found in mRNAs associated with nutritional sensing, carbohydrate metabolism and energy production indicating that yeast RNase III is a key regulator of the cell response to growth conditions. Surprisingly, Rnt1p cleavage sites were not restricted to fixed loop sequence and size but extended to different types of structures that include stems terminating with tri- and penta- loops with varying sequences. The variety and frequency of the cleavage signal suggest that Rnt1p has developed a flexible substrate recognition mechanism capable of discriminating between a wide-range of structured RNAs, while avoiding the cleavage of duplex RNA, which is the classical target of other members of the RNase III family. This unusual substrate specificity explains how a single RNase III may regulate the expression of single RNA under specific condition [14] with high precision, while retaining the flexibility needed for transcriptome surveillance [27].

## Results

### *In silico* queries of NGNN tetraloop structures predict Rnt1p cleavage motifs independent of the RNA context

**Fig 1:**
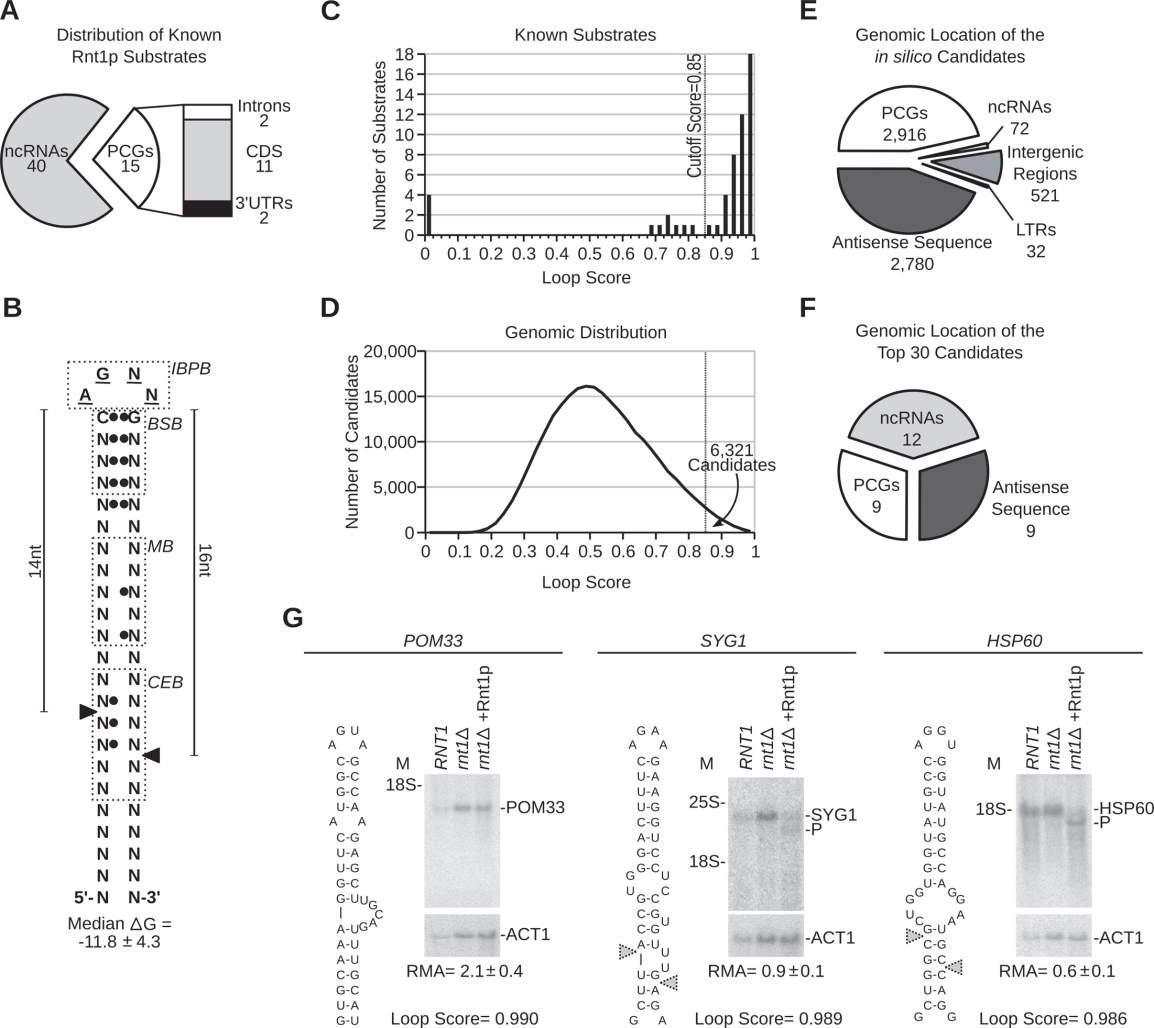
RNA degradation is induced by context dependent activation of randomly distributed cleavage motifs. (**A**) Types of published Rnt1p substrates. Non-coding RNAs (ncRNAs) include snRNAs, snoRNAs and rRNA. Protein coding genes (PCGs) include ORFs, introns and UTRs (see **S1 Table**). (**B**) Schematic representation of the common features of Rnt1p G2-loop substrates. Unpaired nucleotides are underlined. Black circles indicate preferential nucleotide pairing. Enriched bases are named while N indicates any nucleotide. The cleavage sites are indicated by arrowheads. IBPB, BSB, MB and CEB indicate initial binding and positioning box, binding stability box, middle box and cleavage efficiency box [10,37], respectively. (**C**) The score of known substrates was calculated (see **S1 Fig.**) and displayed as histogram. (**D**) G2-loops were scored across the genome and the loop frequency indicated as histogram curve. (**E**) Pie chart illustrating the types of RNA associated with Rnt1p loops. Antisense and LTRs indicates orphan loops located opposite to an annotated gene or found in long terminal repeat elements, respectively. (**F**) Pie chart illustrating the types of RNA harboring the top 30 scoring loops (see **S5 Table**). (**G**) Northern blot analysis of Rnt1p cleavage products. RNA extracted from *RNT1* and *rnt1∆* cells was incubated with recombinant Rnt1p (*rnt1∆* + Rnt1p) and the cleavage products visualized using gene specific probes. ACT1 was used as loading control. The position of the rRNA and products (P) are indicated beside each gel. The relative RNA amount (RMA) was determined using quantitative RT-PCR and indicated below the gels.

There are 55 known substrates of Rnt1p (**Fig. 1A**), the majority of which exhibit a well-defined stem loop structure that features an AGNN tetraloop (G2-loop) (**Fig. 1B**). Therefore, we took advantage of the distinct sequence and structural features of the G2-loop to create an algorithm capable of identifying potential Rnt1p cleavage signals across the entire yeast genome *in silico* (**S1A Fig.**). This algorithm assigns a score (ranging between 0 and 1) to each predicted structure based on sequence conservation, structural stability and similarity to known Rnt1p targets. As shown in **Fig. 1C**, 80% of the known substrates exhibited scores higher than 0.85. On the other hand, substrates not folding into a G2-loop like snR48 or MATa1 intron and those not forming at least three stable base-pairs downstream of the tetraloop (e.g. snR46 and ARN2-1) were given no score. Substrates generated via long-range interaction or based on non-canonical stems (e.g. ADI1, snR59 and snR190) were scored between 0.68–0.81 (**S1 Table**). Accordingly, we chose 0.85 as a cutoff score to retain the majority of known substrates and reduce the number of false positives. Decreasing the cutoff to 0.8 increased the number of detected known substrates by one, while adding 7071 weak hits. On the other hand, increasing the score to 0.9 resulted in the loss of two known substrates and the removal of 4036 putative hits.

Overall, the algorithm identified 254349 possible loops of which 6321 exhibited a score equal to or higher than 0.85 (**Fig. 1D** and **S2 Table**). To validate the reactivity of the predicted cleavage signals and directly evaluate the validity of the cutoff threshold, we synthesized 24 randomly selected stem-loop structures spanning the score range between 0.85 and 1 and tested them for cleavage *in vitro*. The majority of the synthesized loops exhibited scores ranging from 0.85–0.90, which reflect the overall score distribution. To ensure the efficient transcription and structural stability of the different loops we added three G-C base-pairs at the ends of the stems. The added nucleotides are located outside the known binding and cleavage regions and thus, should not affect cleavage efficiency [23,28]. As indicated in **S1B Fig.** and **S3 Table**, the enzyme cleaved all but four of the tested stem-loop structures. The 4 non-reactive stem-loops did not share similar scores but instead featured wobble base pairing (G-U) in the first 2 positions downstream of the loop (S1 Fig. and S4 Table). Based on this result we expect that 83% (with a 95% confidence range of 61 to 95%) of the *in silico* predicted cleavage sites with scores between 0.85 and 1 are cleaved by Rnt1p *in vitro*. Therefore, while the algorithm may falsely recognize a group of non-reactive stem-loops due to the inclusion of inhibitory features, like non-canonical base pairing, the majority of the predicted loops appears to be cleavable by Rnt1p *in vitro*.

Analysis of the genomic distribution of the newly identified G2-loops indicated that 46% reside in protein coding genes (PCGs), 44% opposite to an annotated gene (antisense) and 8.2% in intergenic regions, while only 1.1% were detected in non-coding RNA (**Fig. 1E** and **S4 Table**). This distribution reflects the normal repartition of the yeast genome without preferences to transcript type. On average, potential Rnt1p substrates were distributed in the yeast genome every 4 to 6 kb and displayed similar distribution patterns in other genomes and randomized sequence (**S1C Fig.**). Remarkably, the majority of the predicted cleavage motifs were found in untranscribed regions (e.g. antisense, intergenic regions) confirming that the genomic distribution of the cleavage motifs is not driven by RNA expression (**Fig. 1E** and **S2 Table**). Therefore, the high substrate frequency does not indicate a particularly high demand for RNA degradation in yeast but instead reflects the loose features of Rnt1p substrates.

As expected, examination of the 30 sequences showing the highest score revealed strong enrichment in cleavage signals associated with the processing of pre-snoRNAs (**Fig. 1F**), which constitute the majority of transcripts in the algorithm’s training set. Interestingly, 30% of the top scoring stem-loops were found in mRNAs and 77% of these were located in previously unidentified substrates (**S5 Table**). Cleavage of three of the highest scoring mRNAs was tested *in vitro* (**Fig. 1G**). All three mRNAs (POM33, SYG1 and HSP60) had a loop score > 0.98 and their expression levels varied between 1.3–8.7 copies per cell [29]. Despite the similarity between cleavage motifs, only SYG1 and HSP60 mRNAs were cleaved by Rnt1p suggesting that sequence outside the stem-loop structure may influence substrate reactivity. Consistently, a short RNA transcript corresponding to the POM33 stem-loop was accurately cleaved by Rnt1p *in vitro* when expressed outside its natural mRNA context (**S1D Fig.**). This confirms the accurate prediction of the stem-loop structure and suggests that the lack of cleavage is due to context dependent changes in the stem-loop structure, stability or accessibility. We conclude that RNA degradation in the yeast transcriptome is not limited by the recurrence of Rnt1p cleavage motifs but depends on the surrounding sequence context that influences its formation and reactivity.

### Deletion of *RNT1* perturbs gene expression regardless of the substrate biochemical reactivity

**Fig 2:**
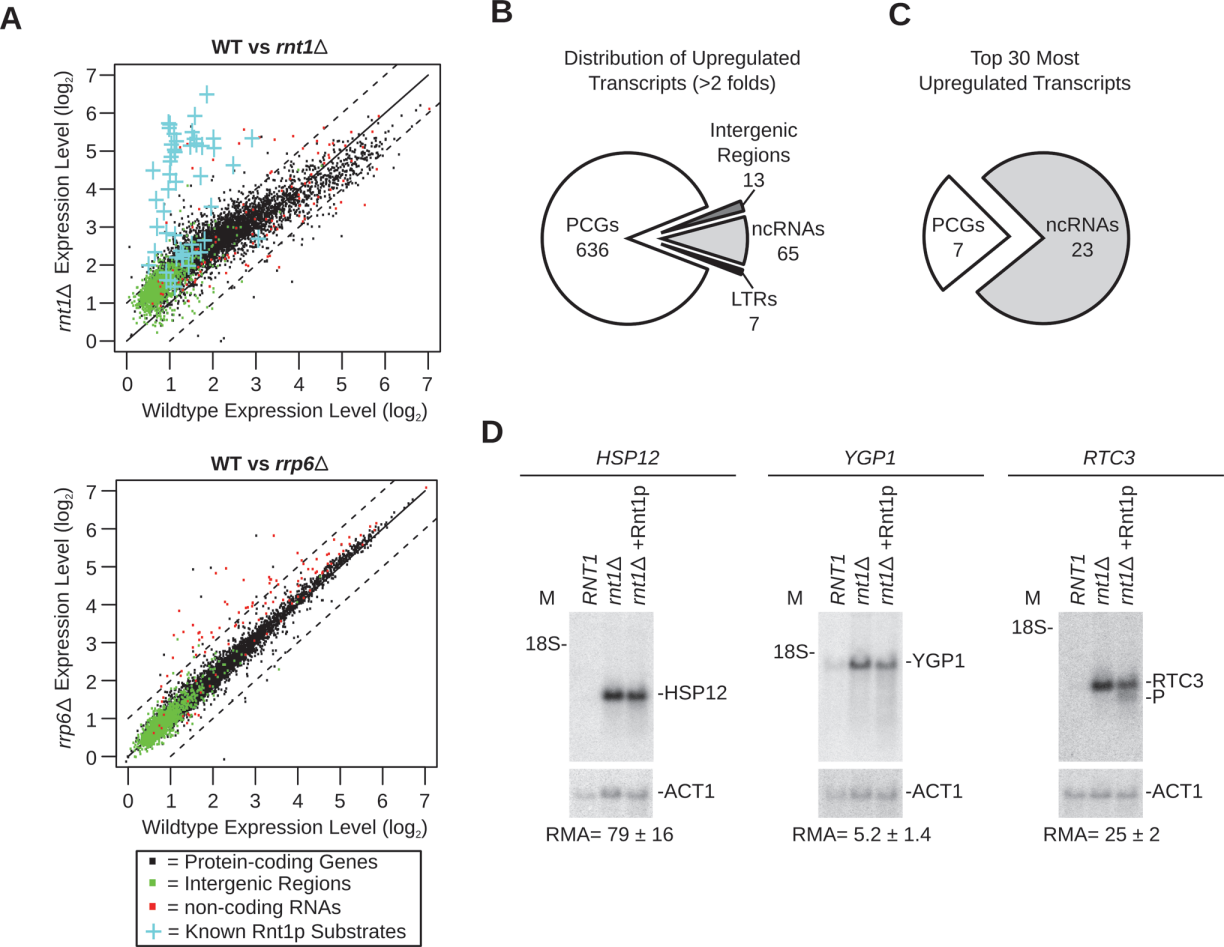
Gene expression is a poor indicator of substrate reactivity. (**A**) Deletion of *RNT1* induces global perturbation of the yeast transcriptome. The levels of gene expression in wild type (WT) cells, *rnt1∆* (top) and *rrp6∆* (bottom) strains were determined using tiling arrays covering the entire yeast genome and presented in the form of dot plot comparison. Dots corresponding to the expression values of protein-coding genes, non-coding RNA and intergenic regions are shown in black, red and green, respectively **(S7 Table)**. Blue crosses highlight the expression of the known Rnt1p substrates as indicated in **Fig. 1A**. (**B**) Rnt1p regulates the expression of both coding and non-coding genes. Pie chart illustrating the types of RNAs upregulated by at least two folds upon *RNT1* deletion (details in **S9 Table**). Multiple transcripts within the same overexpressed segments were counted individually. PCGs, ncRNAs and LTRs indicate protein-coding genes, non-coding RNAs and long terminal repeats, respectively. (**C**) Expression of non-coding RNAs (ncRNAs) is highly sensitive to *RNT1* deletion. The top 30 upregulated genes in *rnt1∆* cells were sorted according to their RNA types (details in **S10 Table**). (**D**) RNAs upregulated by the deletion of *RNT1* resist cleavage *in vitro*. Northern blot analysis of RNA extracted from wild type (*RNT1*) and *rnt1∆* cells before and after incubation with Rnt1p (*rnt1∆*+Rnt1p) was performed as described in **Fig. 1G**. Cleavage was visualized using probes complementary to the sequence of HSP12 (left), YGP1 (middle) or RTC3 (right) mRNAs.

Ribonuclease dependent changes in RNA expression were previously used to identify RNA degradation targets [30,31]. Therefore, we compared the transcriptome of *RNT1* and *rnt1∆* cells using tiling arrays (**Fig. 2 and S2**). The assay was performed once and the data was normalized using unaffected genes, auxotrophic markers or intergenic region as reference or using robust variance stabilization (**S3 Fig.**). The different methods resulted in similar overall data distribution and exhibited comparable statistical confidence when validated using quantitative RT-PCR (**S2C Fig., S3 Fig.** and **S6 Table**). However, normalization using auxotrophic markers resulted in a slightly better Spearman correlation coefficient with the quantitative RT-PCR data and was thus used for further data analyses. Indeed, the auxotrophic markers based normalization method resulted in a correlation factor of 0.798 (p < 2.2E-16, n = 202, which meets the previously established standard for expression array [32]. Moreover, 94.2% of the genes displaying more than two folds increase in the expression array also showed more than 2 folds increase by quantitative RT-PCR (n = 52). Accordingly, we used the 2 folds change in RNA levels as a robust indicator of Rnt1p dependent modification of gene expression.

As expected, the expression of most known Rnt1p substrates increased in *rnt1∆* cells by > 2 folds (**Fig. 2A** upper panel and **S1 Table**). A minority of known substrates was overexpressed between 1.2 and 2 folds and only one (snR66) [33], which is also processed by other ribonucleases, was not upregulated. Overall, 498 segments (overlapping 721 genes) were upregulated by more than 2 folds in *rnt1∆* cells (**S9 Table**). In comparison, only 36 genes (mostly snoRNA genes) were found to be upregulated in the absence of the nuclear exoribonuclease *RRP6* (**Fig. 2A** and S8 Table), The majority of Rnt1p dependent transcripts associated with protein coding genes (**Fig. 2B**), while the majority of the 30 most upregulated sequences in *rnt1∆* cells associated with snoRNA genes (**Fig. 2C** and **S10 Table**). The big difference in the expression of snoRNAs resulted mostly from the retention of the externally transcribed spacers (ETS) that are normally processed by Rnt1p (**S2D Fig.** and [25]). In contrast, none of the 7 most overexpressed mRNAs were previously identified as Rnt1p targets. Northern blot and quantitative RT-PCR analysis of three of those mRNAs confirmed the array predicted overexpression, but only RTC3 was cleaved by Rnt1p (**Fig. 2D)**. We conclude that mRNA overexpression in *rnt1∆* cells does not necessarily predict the enzyme biochemical reactivity but instead mostly identifies genes that are indirectly affected by the deletion of *RNT1*.

### Genome-wide profiling of biochemical reactivity identifies Rnt1p cleavage targets independent of variation in gene expression

**Fig 3:**
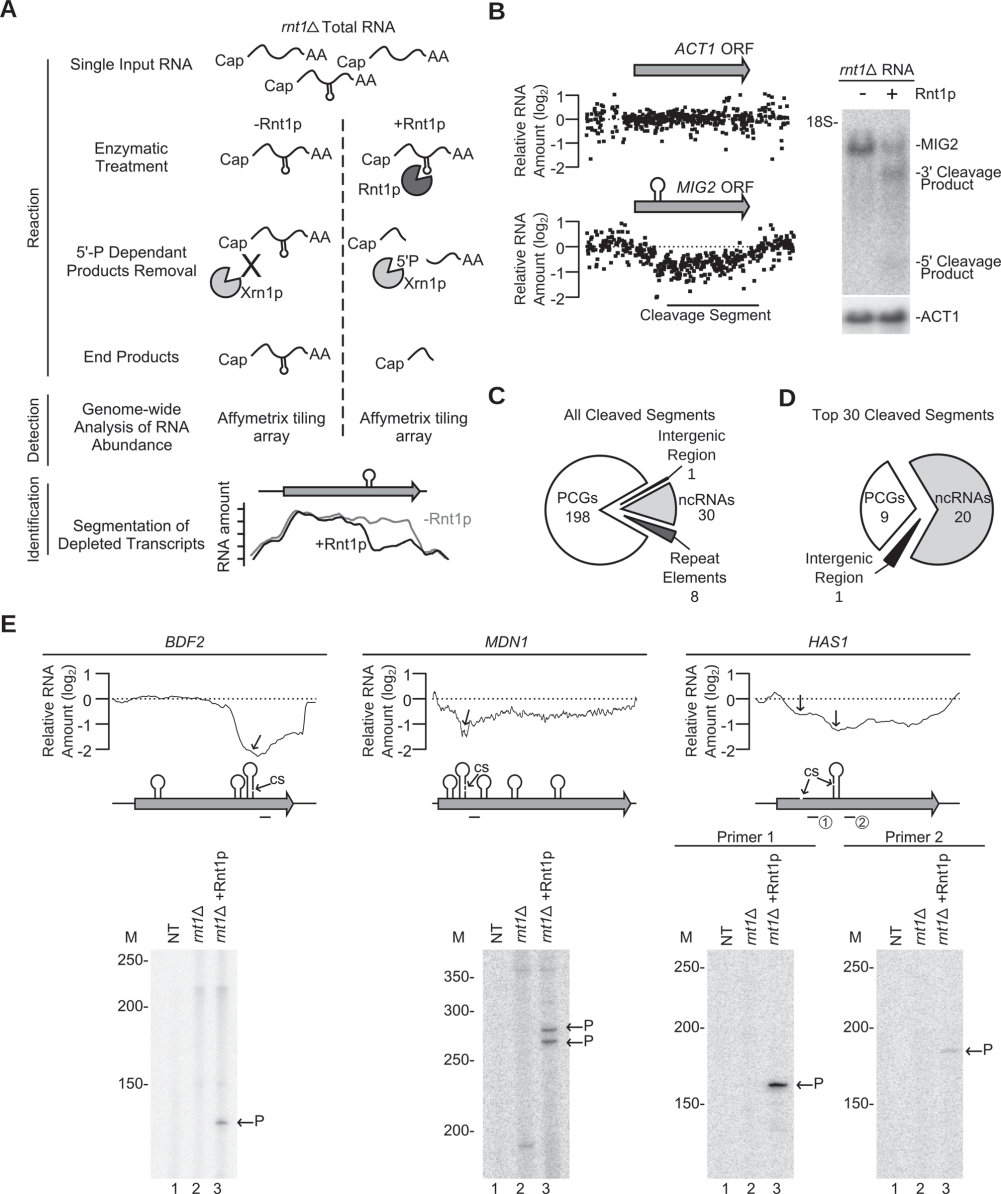
Gene expression independent identification of RNA degradation targets. (**A**) Cut and Chip: a strategy for detecting Rnt1p substrates using tiling array. *rnt1∆* RNA was extracted, cleaved with recombinant Rnt1p and the cleavage products degraded using Xrn1p. Differences between the treated and untreated RNA was detected using Affymetrix tiling array and the cleavage fragments identified (**S4 Fig.**). (**B**) Cut and Chip accurately identifies Rnt1p targets. Comparison between the Mig2 mRNA degradation pattern [13] detected by Cut and Chip (left panel) and Northern blot (right panel). ACT1 mRNA was included as negative control [13]. The relative levels of RNA detected by the different probes (black dots) are presented below each ORF. The cleavage sites are shown as stem-loops and the cleaved segments indicated by black line. The Northern blot was performed as described in **Fig. 1G**. (**C**) Pie chart illustrating the types of RNA cleaved by Rnt1p (**S11 Table**). (**D**) Pie chart illustrating the type of the top 30 cleaved RNAs (**S12 Table**). (**E**) Identification of Rnt1p cleavage site using primer extension. Reverse transcription was performed using gene specific radiolabelled primers before (*rnt1*∆) and after (*rnt1*∆ + Rnt1p) cleavage. NT, M and P indicate no template control, size markers, and the cleavage product 5’ end, respectively. The smoothed cleavage profile is shown relative to the ORF on top. The predicted G2-loops are indicated as stem loops and the detected cleavage site (CS) identified by the arrows. Probes used for primer extension are shown below each ORF.

Detection of catalytic activity is the best and most direct way to uncover the substrates of any enzyme. Therefore, we have developed a new method termed “Cut and Chip” that permits direct detection of all the RNAs cleaved by Rnt1p in the yeast transcriptome (**Fig. 3A and S4 Fig.**). In this new method, the 3’ end cleavage products generated by Rnt1p *in vitro* are degraded by the 5’-3’ exoribonuclease Xrn1p [34] and the decrease in the RNA level is detected using tiling array (**S4A Fig.**). As shown in **Fig. 3B**, 50% cleavage of Rnt1p substrate (MIG2) [13] was easily detected by the decrease in the array signals downstream of the cleavage site in both Rnt1p and Xrn1p dependent manner. Overall, this approach detected cleavage in 237 RNA transcripts (**S11 Table**), which represent 4% of the yeast genes. Cleavage motifs were detected in 79% of the cleaved RNA (**S4C Fig.**) suggesting that the majority of Rnt1p substrates uses G2-loops for cleavage. The majority (71%) of the 55 known substrates were positively identified in this assay. However, we found that detection of cleavage events was dependent on the length of the transcribed sequence downstream of the cleavage site (i.e. length of the 3’ end product) and strength of cleavage (**S4D Fig.)**. Therefore, weak cleavage events and those producing 3’ end fragments smaller than 50 nucleotides may not be detected by this technique. Interestingly, cleavage of native RNA by Rnt1p in whole cell extracts produced similar results to that detected by the cleavage *in vitro* (**S11 Table**). We conclude that the reactivity of the majority of Rnt1p targets (~80%) *in vitro* is not significantly modified through the RNA extraction process or concealed by other protein factors. However, it remains possible that the reactivity of certain RNA is affected by cellular compartmentalization or requires other *in vivo* events such as active transcription. Nonetheless, the results suggest that, at least *in vitro*, Rnt1p cleaves at least four times more transcripts than previously demonstrated.

The majority (83%) of the cleaved segments were found in coding sequence (**Fig. 3C** and **S11 Table**). Interestingly, 9 out of the 12 genes longer than 7.5 kb in the yeast genome were efficiently cleaved by Rnt1p (**S11 Table).** Also, only 8 cleavage events were found in introns and the majority of these (7/8) degrades the intron of mRNAs coding for RNA binding proteins. These introns did not encode for snoRNAs suggesting that cleavage in these pre-mRNAs is not part of a processing pathway. The top 30 genes cleaved by Rnt1p included 18 known substrates, 5 new non-coding RNA substrates (e.g. snR85, snR60, snR81, U3b and TLC1) and 7 new mRNAs, 3 of which code for genes associated with ribosome biogenesis (HAS1, MDN1 and BFR2) (**Fig. 3D** and **S12 Table**). Primer extension analysis of three newly identified substrates confirmed the capacity of “Cut and Chip” to accurately detect Rnt1p reactivity *in vitro* (**Fig. 3E)**. However, primer extension also indicated that “Cut and Chip” does not accurately identify the precise site of cleavage. In most cases, the cleavage segment boundary was associated with several G2-loops and did not always coincide with the position of the cleavage site (**Fig. 3E**). This observation was further validated by primer extension of 3 additional “Cut and Chip” predicted substrates (**S4E Fig.**). Therefore, while “Cut and Chip” is a strong predictor of Rnt1p RNA targets *in vitro*, it does not directly identify the sequence of the cleavage site.

### Sequencing of Rnt1p cleavage products reveals unexpected diversity of substrates structure

**Fig 4:**
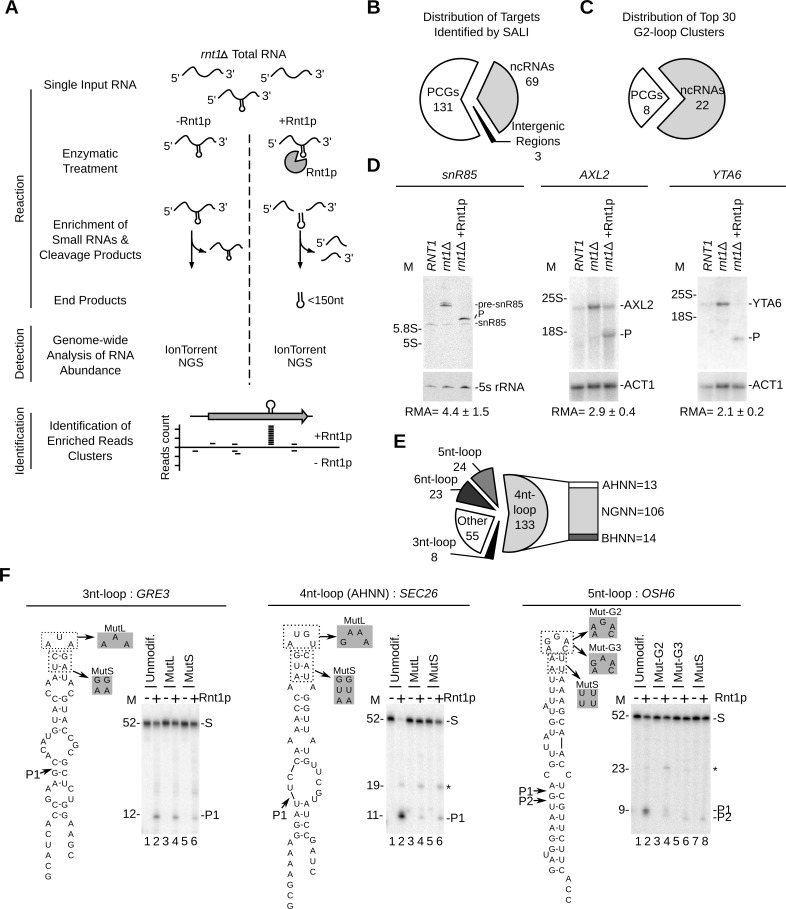
Sequence assisted identification of Rnt1p cleavage signals. (**A**) Strategy for Sequencing Assisted Loop Identification (SALI). In this strategy, *rnt1∆* RNA is cleaved by Rnt1p, RNA fragments < 150 nucleotides enriched and the 32–38 nucleotides internal cleavage fragments identified using next generation sequencing (NGS) (**S5 Fig.**). (**B**) Pie chart illustrating the types of RNAs associated with at least one enriched read cluster (details in **S15 Table**). (**C**) Distribution of the top 30 enriched read clusters by RNA type (details in **S16 Table**). (**D**) Cleavage of new substrates was verified using Northern blot as described in **Fig. 1G**. ACT1 mRNA and 5S rRNA were included as negative controls. The relative RNA expression (RMA) was calculated as described in **Fig. 1G**. (**E**) SALI uncovers new classes of Rnt1p substrates. The cleavages products identified by SALI were folded and the resulting structures shown in the form of pie chart. Unfolded RNAs and stems capped with loops larger than 6 nucleotides were classified as “other”. The loop sequence is described using standard single letter nucleotide code (IUPAC). (**F**) Validation of the new classes of Rnt1p substrates. A representative member of each loop type (unmodif) as well as mutated loop (MutL) and stem (MutS) versions were transcribed and cleaved by Rnt1p. The cleavage products were identified using 20% denaturating PAGE (see also **S5E Fig.**). Substrate (S) and products (P) are indicated on the right. The size markers are indicated on the left. The observed cleavage sites are indicated by arrowheads.

To directly detect Rnt1p cleavage site, we developed a Sequencing Assisted Loop Identification (SALI) technique that permits direct identification of Rnt1p cleavage product (**Fig. 4 and S5 Fig.**). In this method, the internal cleavage fragment released by Rnt1p is directly sequenced permitting the identification of reactive cleavage signal (**Fig. 4A**). An average of 4.2 million sequencing reads were obtained from both control and cleaved RNA and the 32–38 nucleotides-long reads enriched in the cleaved samples were retained (**S5B Fig.**). As expected, the cleaved RNA sample exhibited a net enrichment in reads ranging between 32 and 38 nucleotides, while most of the reads detected in the control RNA were mapped to abundant small RNAs shorter than 150 nucleotides like tRNAs and snoRNAs. Overall, this technique identified 34 out of 55 known Rnt1p cleavage signals (**S13 Table**). Notably, the boundaries of the enriched reads clusters matched almost perfectly with the position of the cleavage sites reported in the literature (**S5C Fig.**). The missing substrates were either poorly cleaved (e.g. HSL1) [26], produced products longer than 38 nucleotides (e.g. snR51) or were expressed at low levels (e.g. RGT1) [14]. The false positive rate of this technique is estimated to be < 7% based on a list of 30 mRNAs, which showed no cleavage by Northern blot (**S14 Table**). Therefore, SALI is a robust tool for the direct detection of highly reactive Rnt1p cleavage sites.

Overall, SALI detected 243 enriched read clusters corresponding to 203 unique targets (**S5D Fig.** and **Tables S13** and **S15**). The cleavage sites were associated with 131 protein-coding genes, 69 non-coding RNA genes and 3 intergenic regions (**Fig. 4B)**. The 30 most enriched cleavage products were found associated with 22 non-coding RNAs and 8 mRNAs (**Fig. 4C** and **S16 Table**). The most enriched sequence mapped to the 5’ETS of a previously uncharacterized cleavage site near the H/ACA snoRNA snR85. Northern blot analysis confirmed the cleavage of the snR85 precursor, which accumulates in *rnt1∆* cells (**Fig. 4D** left panel). The 8 highest cleaved mRNAs included only 1 known substrate (MIG2) [13] and 7 new targets (HSP60, TUB1, AXL2, MAP2, HXK1, YTA6 and NAR1) (**S16 Table**). Northern blot analysis of three of these RNAs (HSP60, AXL2 and YTA6) confirmed the *in vitro* cleavage predicted by SALI. In addition, both AXL2 and YTA6 mRNAs accumulated in *rnt1∆* cells indicating that these substrates require Rnt1p for normal expression.

Surprisingly, only 44% of the newly identified cleavage products formed the NGNN tetraloop structures deemed essential for Rnt1p reactivity (**Fig. 4E** and **S13 Table**). The rest of the cleavage fragments were either unfolded or formed non-canonical stem loop structures. The newly identified structures included stems capped with either AHNN and BHNN tetraloops or loops with sizes varying between 3 and 6 nucleotides (**Fig. 4E)**. To verify the reactivity of these new structures, we generated T7 RNA polymerase transcripts representing the different loop structures and tested them for cleavage *in vitro*. Structures exhibiting AHNN tetraloops, pentaloops, or triloops were successfully cleaved by Rnt1p while those exhibiting BHNN and hexaloops displayed poor or no reactivity (**Fig. 4F and S5E Fig.**). Mutations of the AHNN, pentaloops and triloops indicate that both the structure and sequence of the new loops are required for optimal cleavage (**Fig. 4F and S5E Fig.**). Notably, replacement of the established U5 snoRNA G2-tetraloop with the newly identified pentaloop structure of OSH6 did not affect Rnt1p cleavage (**S5F Fig.**). This indicates that pentaloop and G2-loop have comparable reactivity and confirm the newly identified structure as robust Rnt1p substrate. Moreover, Rnt1p cleavage was also observed in the host transcripts of SEC26 and OSH6, further confirming their capacity to be cleaved by Rnt1p (**S5G Fig.**). We conclude that Rnt1p substrate selectivity *in vitro* is not limited to NGNN tetraloop, but extends to a broad range of structured RNAs, which can be distinguished from generic RNA duplexes that are not cleaved by Rnt1p [22].

### Comparison and validation of the newly identified RNA degradation targets

**Fig 5:**
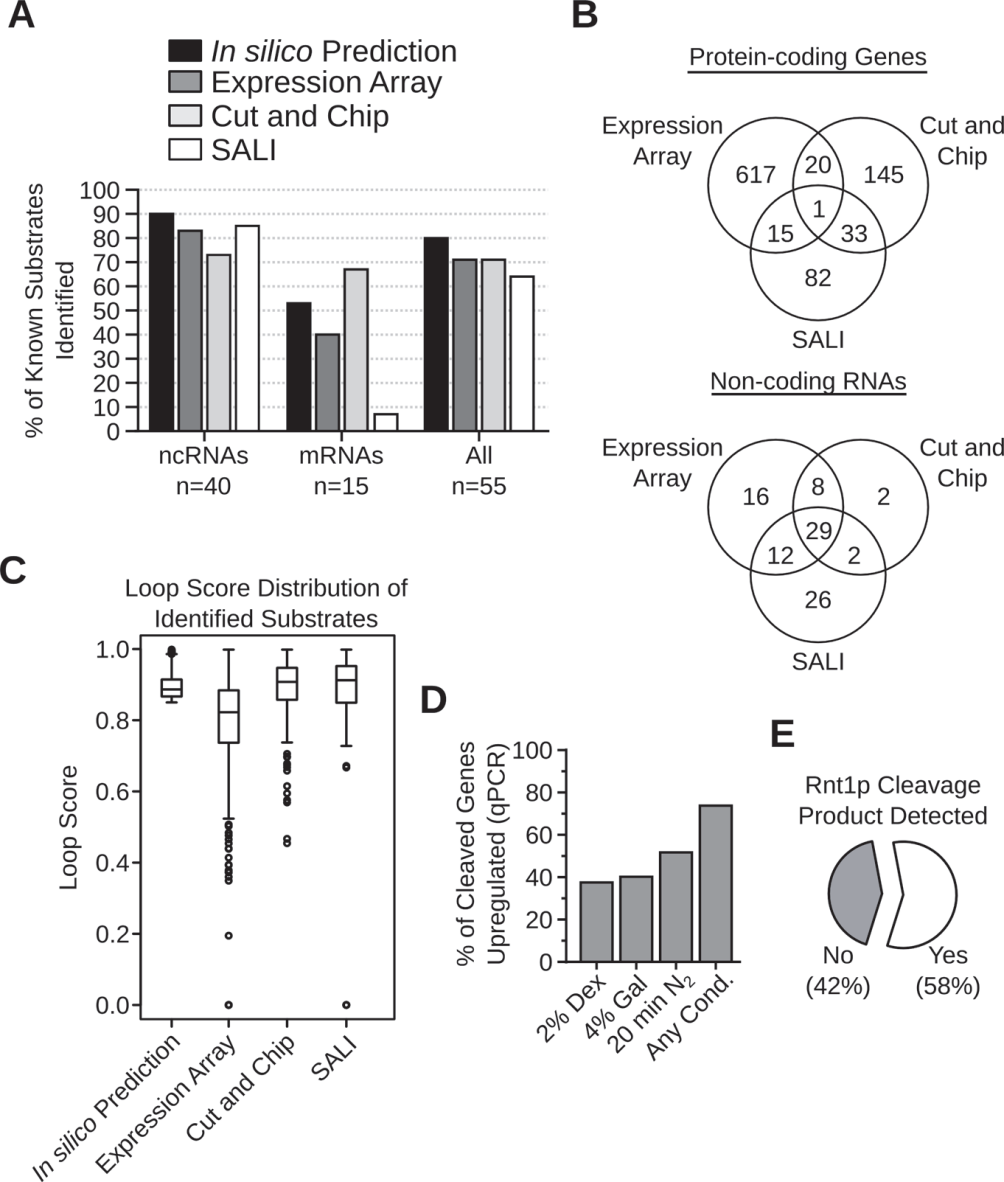
Evaluation of the different methods used for the detection of Rnt1p cleavage targets. (**A**) Bar graph indicating the percentage of known substrates (**Table S1**) detected by each substrate detection assay. (**B**) Venn diagrams showing the number of Rnt1p cleavage targets identified by the different detection methods in protein-coding genes (top panel) or non-coding RNAs (bottom panel). (**C**) Box plots showing the distribution of the loop scores associated with the targets identified by each detection method. (**D**) Bar graph showing the percent of Rnt1p *in vitro* cleavage targets upregulated after the deletion of *RNT1* under different growth conditions. The RNA was extracted from *RNT1* and *rnt1∆* cells grown in media containing dextrose (2% Dex) or galactose (4% Gal) or cells shifted for 20 minutes in nitrogen supplemented media (20min N_2_). The expression levels were examined by quantitative RT-PCR using primers complementary to 109 *in vitro* cleavage targets not detected by the expression array (see **S17 Table**). Genes showing > 1.2 folds difference in expression between *rnt1∆* and *RNT1* cells with p-value < 0.01 were considered upregulated. The presented data are the average of three independent experiments. (**E**) Pie chart showing the percentage of Rnt1p cleavage sites associated with the accumulation of 5’-P cleavage products in *xrn1∆ / dcp2∆* cells [35].

The candidate substrates generated by the computational analysis, expression array, Cut and Chip and SALI were compared to evaluate the merit of each method. As indicated in **Fig. 5A**, all four methods were able to detect 65–80% of all known substrates and, in general, were better at detecting non-coding RNAs. The computational approach identified the highest number (90%) of the known non-coding RNA targets, which were used as training set for the algorithm, while Cut and Chip identified the highest number (67%) of the known mRNA targets. SALI performed worst at identifying known mRNA targets, presumably because SALI is more dependent on the expression level of the transcripts. Indeed, among 9 known mRNA substrates which were not detected by SALI, 7 (78%) are expressed at less than 1 copy / cell (**Table S1**).

Comparison between the results of the expression array, the Cut and Chip and SALI revealed little overlap between the newly identified RNA targets (**Fig. 5B**). Only 1 mRNA and 29 (31%) non-coding RNA targets were detected by all three methods. The lowest overlap was found between the *in vitro* cleavage (Cut and Chip and SALI) and the expression-based assays (**Fig. 5B**). Analysis of the stem-loop scores associated with RNA identified by each of the three detection methods indicated that, in general, the RNA identified by the expression array have low loop scores while the highest loops scores were found in RNA identified by the *in vitro* cleavage assays (**Fig. 5C**). This suggests that the RNA identified by the expression array have less potential to carry a reactive cleavages signal than those found with the *in vitro* cleavage assays. Indeed, out of the 653 RNAs detected by the expression array, only 36 were cleaved *in vitro* (**Fig. 5B**), suggesting that the majority of these RNAs are indirectly affected by the deletion of *RNT1*. On the other hand, a large proportion of the *in vitro* cleavage targets were not identified by the expression array, likely due to the limited sensitivity and the growth conditions. Indeed, several studies show that Rnt1p can affect the expression of its targets in a condition dependent manner [14,26]. Thus, the newly found targets may not accumulate in absence of *RNT1* when cells are grown in optimal conditions. To directly evaluate this hypothesis, we tested the expression of 109 randomly selected *in vitro* cleavage targets that were not identified by the expression array using quantitative RT-PCR under three different growth conditions (**S17 Table**). As indicated in **Fig. 5D**, 74% of the tested RNAs were upregulated (>1.2 folds and p-value <0.01) by the deletion of *RNT1*, suggesting that the majority of the cleavage targets are inhibited by Rnt1p *in vivo*. However, the upregulation of many targets was detected only under specific growth conditions, suggesting the Rnt1p expression is required for condition dependent repression of gene expression. Notably, the cleavage product of 57% of the highly expressed *in vitro* cleavage sites could be detected *in vivo* upon the deletion of *XRN1*, which normally degrades Rnt1p cleavage products (**Fig. 5E**) [27,35]. Together these data supports the *in vivo* reactivity of the newly identified cleavage signals.

### Rnt1p cleaves stem-loop structures with different sequence and base pairing requirements

**Fig 6:**
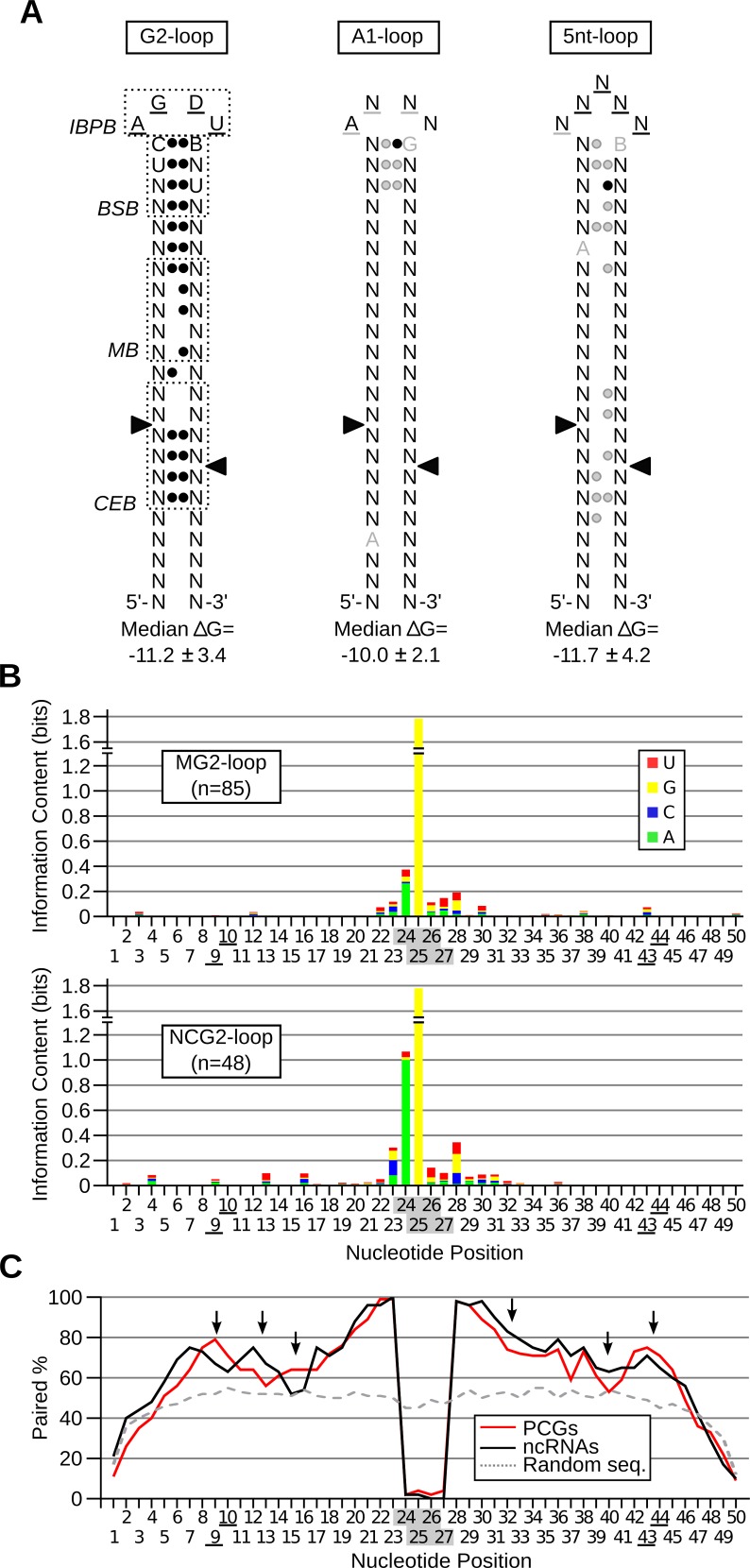
Rnt1p cleaves different classes of stem-loop structure with diverse sequence and structural requirements. (**A**) Revised model of Rnt1p cleavage signals illustrating the common features of reactive RNAs. The substrates were classified based on the loop size and sequence and the three classes exhibiting statistically significant features (p-value < 0.05) were illustrated in the form of stem-loop structures. Underlined nucleotides indicate unpaired positions; black circles indicate paired positions. Grey underline, nucleotide or circle indicates the features, which are frequently observed, but not statistically enriched. Arrowheads indicate the cleavage sites. IBPB, BSB and CEB indicate initial binding and positioning box, binding stability box, and cleavage efficiency box [10,28], respectively. The median Gibbs energy (∆G) was also calculated within each group of substrates. (**B**) The processing and degradation signals display different sequence preferences. The sequence of G2-loop substrates required for protein-coding mRNA degradation (MG2-loop; top panel) was compared to those involved in non-coding RNA processing (NCG2-loop; bottom panel) and the information content of each nucleotide position illustrated in the form of a composite bar graph. The shaded and underlined numbers indicate the position of the tetraloop and cleavage sites, respectively. (**C**) Rnt1p cleavage sites are preferentially base-paired in protein coding substrates. The percent paired nucleotides at each position of Rnt1p cleavage signals was determined in non-coding RNAs (ncRNA), protein-coding genes (PCGs) and randomly generated sequences (Random seq) and presented in the form of a line graph. Arrows indicate differences between the two groups of cleavage signals.

The large number of new substrates identified during this study permits better definition of Rnt1p substrates. Sequence comparison of the G2-loop substrates indicated that the ideal consensus sequence of the G2-loop is AGDU (**Fig. 6A** left panel), confirming the high conservation of the first two nucleotides and suggests that the 3^rd^ and 4^th^ nucleotides of the loop might be also important for the enzyme reactivity. This finding is supported by earlier work indicating that the enzyme interacts and forms hydrogen bonds with these two nucleotides [28,36]. In addition, comparison of the stem sequence revealed preference in the nucleotide adjacent to the loop, which was previously shown to affect cleavage [10,22,36]. The new model of G2-loop also indicated preference for base pairing in the first 7 nucleotides downstream of the tetraloop consistent with previous biochemical studies indicating the requirement of the first 5 base pairs for cleavage by Rnt1p [37]. Unlike the G2-loops, only a slight tendency to base pairing was detected near the A1- and 5nt-loops (**Fig. 6A** middle and right panels). The small number of candidates and high variability in sequence and structures of these new classes of substrates limited our ability to detect statistically enriched features. However, in general all classes of Rnt1p substrates displayed a tendency to form stable structures with an apparent Gibbs energy below -10.0 Kcal/mol.

Comparison between the Rnt1p G2-loops required for the processing of non-coding RNAs (NCG2-loop) to those required for mRNA (MG2-loop) degradation revealed few differences in sequence and structure. Uracil is preferred in the third position of the NCG2-loops, while guanine is predominant at this position of MG2-loops (**Fig. 6B**). Surprisingly, the most marked difference between the two groups of G2-loops was the stem base pairing preferences (**Fig. 6C**). The nucleotides near the cleavage sites (positions 9, 10, 43 and 44 in **Fig. 6C**) and those in the middle stem (positions 15 and 16) were preferentially unpaired in NCG2-loops. The increased mispairing in the more reactive NCG2-loop suggests that unpaired cleavage sites increase reactivity. Indeed, forced pairing of these nucleotides within the cleavage efficiency box decreased the catalytic rate without affecting the substrate affinity [10].

### Rnt1p preferentially cleaves mRNA associated with carbohydrate metabolism and respiration

**Fig 7:**
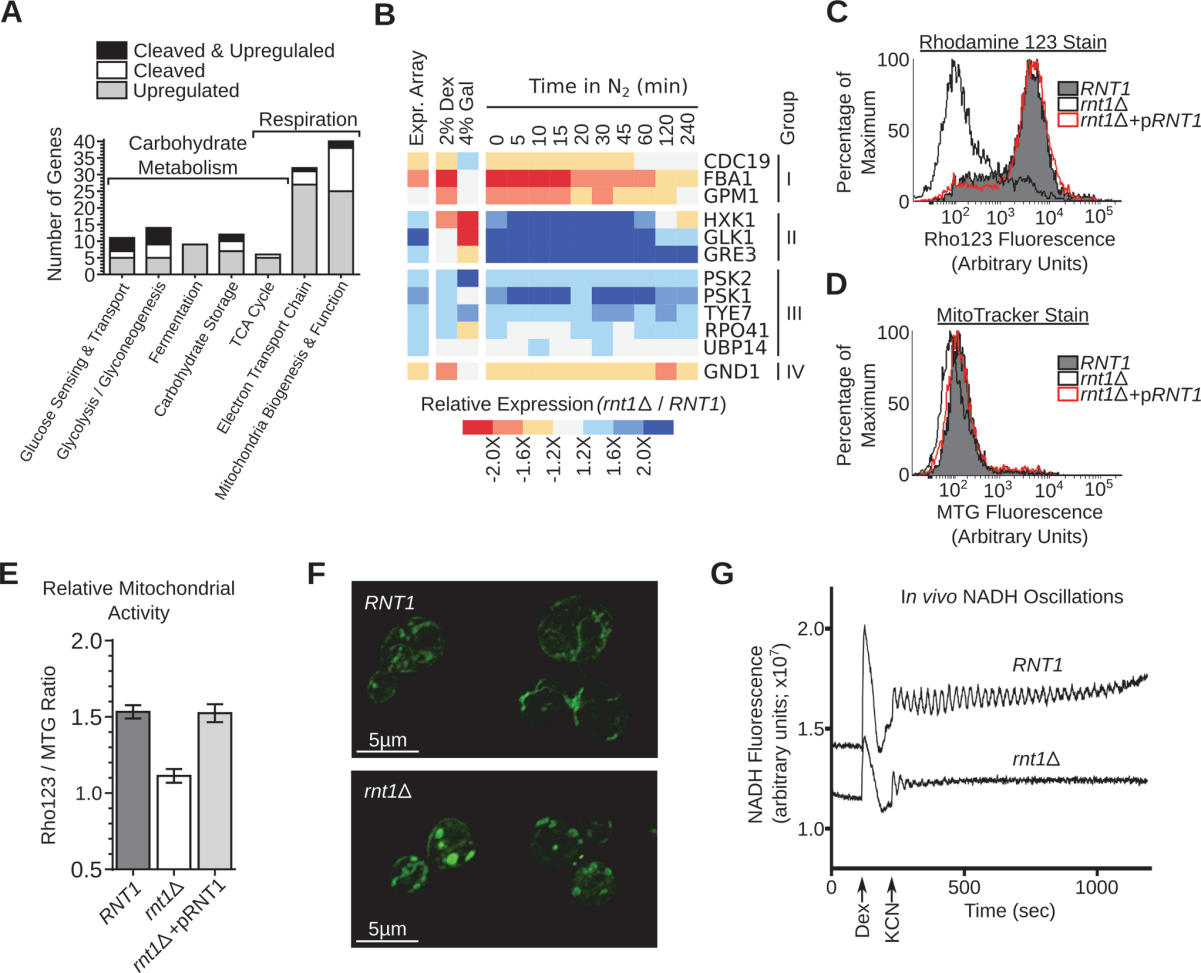
Deletion of *RNT1* impairs the expression of *in vitro* substrates associated with respiration and carbohydrate metabolism. (**A**) RNA degradation signals accumulate in genes associated with carbohydrate metabolism and energy production. Genes affected by Rnt1p were classified using gene ontology (GO) [69], MIPS database [39] and literature search (**S18 and S19 Table**) and those related to respiration and carbohydrate metabolism shown in the form of a bar graph. (**B**) The expression of Rnt1p substrates was examined in *RNT1* and *rnt1∆* cells grown on dextrose (2% Dex), galactose (4% Gal) or after different time following the shift from oxygen to nitrogen supplemented media (N_2_). The relative expression levels were determined using microarray (Expr. Array) or quantitative RT-PCR and presented in the form of heat-map (see also **S6 Fig.**). Genes are grouped according to their response to oxygen depletion. The data shown are an average of three biological replicates. (**C**) *RNT1* and *rnt1∆* cells transformed with either the empty vector or pRS316 expressing the wild type *RNT1* allele (pRNT1) were stained with Rhodamine 123 (Rho123) and analyzed by flow cytometry. (**D**) *RNT1* and *rnt1∆* cells were stained using the Mitotracker stain (MTG) and analyzed by cytometry. (**E**) The ratio of the Rho123 to MTG signals were calculated for each strain and presented in the form of a bar graph. (**F**) *RNT1* (top) and *rnt1∆* (bottom) cells were stained with Mitotracker and the mitochondria (shown in green) visualized using epifluorescence. (**G**) Rnt1p is required for glycolytic oscillations. Glucose-depleted cell suspensions were supplemented with dextrose (Dex) and potassium cyanide (KCN) to induce oscillations and NADH fluorescence was recorded over time using a temperature-controlled spectrofluorometer.

The function of genes either upregulated or cleaved *in vitro* by Rnt1p was examined using gene ontology [38], MIPS database [39] and literature search (S18 and S19 Table). The results indicated that several genes are associated with mitochondrial respiration and carbohydrate metabolism (**Fig. 7A**). Accordingly, we monitored the effects of variation in carbon sources and oxygen levels on the expression of Rnt1p substrates. As shown in **Fig. 7B and S6A Fig.**, 8 substrates were repressed by the enzyme in dextrose, while 3 were repressed in galactose (CDC19, PSK2 and TYE7). Interestingly, three genes which were not affected or downregulated in absence of *RNT1* in aerobic condition (*CDC19*, *FBA1* and *GPM1*), showed clear differences in their expression pattern in response to the nitrogen shift (**S6B Fig.**). Together, these data indicate that the switch from respiration to fermentation modifies the expression of a subset of conditionally expressed genes in a Rnt1p dependent manner.

To evaluate the effect of Rnt1p on respiration, we monitored the levels of mitochondrial membrane electrical gradient (∆Ψm) using the Rhodamine 123 stain [40]. As shown in **Fig. 7C**, deletion of *RNT1* reduced staining suggesting that the enzyme is required for normal respiration. The change in respiration was not due to changes in the number of mitochondria as indicated by the MitoTracker stain (**Fig. 7D**). Transformation of *rnt1∆* cells with a plasmid expressing a wild-type allele of *RNT1* (*pRNT1*) completely restored the Rhodamine staining to its normal levels confirming the requirement of *RNT1* expression for normal respiration (**Fig. 7C-E**). Consistently, epifluorescence imaging indicated that the morphology of mitochondria was altered in *rnt1∆* cells (**Fig. 7F**). Interestingly, despite the perturbation of both fermentation and respiration genes (**S18 and S19 Table**), *RNT1* deletion did not block growth in either state, but reduced growth in all carbon sources (**S6C** and **S6D Fig.**). Therefore, Rnt1p repression of gene expression is not essential for either respiration or fermentation but instead appears to be needed fine-tuning gene expression between different metabolic states. To evaluate this possibility, we examined the effect of *RNT1* deletion on autonomous oscillation. Autonomous oscillations in the concentrations of glycolytic intermediates like NADH reflect the dynamics of control and regulation of this major metabolic pathway required for both respiration and fermentation [41]. Oscillations of both *RNT1* and *rnt1∆* strains was induced by the addition of glucose and potassium cyanide and recorded as time traces of NADH fluorescence (**Fig. 7G**). As expected, oscillations were clearly observed in *RNT1* cells and lasted for about 15 minutes. In contrast, *rnt1∆* cells showed weaker response to glucose induction and oscillations ceased just a few seconds after induction. These results indicate that Rnt1p expression is required for glycolytic oscillations and the efficient coordination of metabolic flux in yeast.

## Discussion

In this study, we used *in silico* (Fig. 1), genetics (Fig. 2) and biochemical methods (Fig. 3 and 4) to define the characteristics and genomic locations of RNA degradation signals. The results indicate that while potential Rnt1p cleavage motifs are evenly distributed across the yeast genome, only few induce the degradation of the host transcript (Fig. 1 and S2 Table). The abundance of Rnt1p recognition motifs suggests that RNA degradation is not limited by the *de novo* evolution of the cleavage motif but instead controlled by the overall structure of RNA transcripts. This is supported by the fact that non-reactive cleavage signals may become reactive in different sequence and structural contexts (**S1 Fig.**). Therefore, while Rnt1p cannot cleave generic RNA duplexes like other members of the RNase III family [22], it retains broad substrate specificity by recognizing simple and widely distributed motifs. This flexible substrate specificity may explain how *S*. *cerevisiae* maintained its symbiotic relationship with the dsRNA killer virus, which confers selective advantage by producing a toxin that kills uninfected strains [42], without limiting the transcriptome surveillance functions of RNase III.

Expression profiling techniques are extensively used to probe the effects of different ribonucleases on RNA stability and gene expression. These techniques permitted the identification of new classes of unstable transcripts like the Rpr6p-dependant cryptic unstable transcripts (CUTs) [43] and Xrn1p-sensitive unstable transcripts (XUTs) [44]. However, the difficulty in distinguishing between the direct and indirect effects of ribonuclease deletions prevented positive identification of catalytically reactive substrates. Indeed, in this study, the results indicate that most transcripts upregulated after *RNT1* deletion resisted cleavage *in vitro* (**S20 Table**). Biochemical assays like PARE (parallel analysis of RNA ends) were developed to identify the degradation targets of miRNAs [45]. In this study, we used analogous approaches that depend on microarray detection (Cut and Chip) and direct sequencing (SALI) for the identification of Rnt1p cleavage products. Both methods accurately identified the majority of the known Rnt1p substrates (**Fig. 5A**) and the newly identified cleavage events were confirmed using standard cleavage assays. These two methods identified distinct sets of Rnt1p substrates (**S20 Table and Fig. 5B**). Cut and Chip mostly detected long 3’ end cleavage products while SALI detected the cleavage of well-defined and highly expressed cleavage signals (see **Figs. 5C** and **S7**). For example, SALI detected the highly expressed snR62 cleavage signal, while Cut and Chip detected the long internal structure of snR51. In short, Cut and Chip is more effective in detecting poorly cleaved RNA with long 3’ end cleavage fragment, while SALI is more efficient in directly detecting the site of cleavage of highly expressed and highly reactive substrates. It should also be noted that both methods might fail to detect substrates expressed at very low levels or requiring special factors not present *in vitro*. Therefore, identification of RNA degradation signals may not be achieved by a single technique but requires a number of complementary approaches that together may provide a true portrait of enzymatic reactivity.

Comparison between the *in vitro* cleavage assay and expression profiling data indicates that the number of RNA that are both cleaved by Rnt1p and overexpressed after the deletion of *RNT1* is very small. Indeed, only 36 out of the 296 mRNA cleaved by Rnt1p *in vitro* are upregulated upon the deletion of Rnt1p *in vivo* as predicted by the tiling array. This discrepancy reflects the effect of the growth condition tested and the limitation of the expression profiling techniques (**S7 Fig.**) that requires arbitrary cutoffs and complicated statistical analysis [38,46–48]. Indeed, we clearly show that variations of the growth conditions and the use of quantitative RT-PCR substantially increase the number of the *in vitro* cleavage targets affected by *RNT1* deletion *in vivo* (**Fig. 5D and S6 Table**). Therefore, it appears that the *in vitro* cleavage assays are better indicators of RNA degradation than the expression array. This is supported by the cleavage of native RNA in cell extracts (**S11 Table**), the detection of many cleavage products *in vivo* (**Fig. 5E**), the reduced potential of expression array candidates to carry reactive cleavage signals (**Fig. 5C**) and the fact that many of the most overexpressed transcripts *in vivo* could not be cleaved *in vitro* (**Fig. 2D** and **S20 Table**). However, we cannot rule out the possibility that the reactivity of certain RNAs might be artificially modified *in vitro* due to the absence of specific *in vivo* conditions like active transcription and cellular compartmentalization.

The NGNN tetraloops (G2-loop) are required for the cleavage of most known Rnt1p substrates [49]. For the selection of G2-loops, the enzyme uses a double-ruler mechanism [28]. In this study, we extended the AAGU tetraloop (A1-loop) into the AHNN category and identified two new categories of Rnt1p substrates that feature triloops and pentaloops (**Fig. 4E**). The mechanism by which the enzyme recognizes these three categories of substrates remains unclear. Mutational and biochemical analysis of substrates carrying G2- and A1-loop indicate that Rnt1p use a flexible and interchangeable network of nucleotide interactions to identify its substrates with different structures [22,24]. This flexibility may also explain how the enzyme may cleave different structures like triloops and pentaloops. Alternatively, the enzyme may induce fit the new loops for binding and cleavage [50]. In all cases, the discovery of these new substrates indicates that Rnt1p activity is not restricted to a single stem-loop structure but cover a much larger spectrum of structural motifs.

In nature, yeast cells are in constant flux between respiration and fermentation depending on sugar and oxygen levels [51]. These constant changes in the cell’s metabolic state require a highly responsive and dynamic control of gene expression [52,53]. In high concentration of glucose, yeast cells prefer fermentative metabolism to oxidative pathway regardless of oxygen levels [54]. This reversible respiro-fermentative metabolic state is characterized by induction of genes involved in both glucose transport and glycolysis [55] and repression of the TCA cycle and mitochondrial activity [56]. Interestingly, this combined induction of both transport and glycolysis genes were also observed upon deletion of *RNT1*. Indeed, glucose sensing (e.g. *MIG2*, *MTH1* and *RGT1*) [14], glucose transport (e.g. *HXT9*, *HXT11*, *HXT13* and *HXT15*), glycolysis (e.g. *FBP26*, *GLK1*) and electron transport chain genes (*QCR7–9* and *CYT1*) were all upregulated in *rnt1∆* cells (**S7 Table**). Overall, there are now a total of 15 genes in these pathways known to be cleaved by Rnt1p *in vitro* and their expression is deregulated by its deletion *in vivo* (**Fig. 7** and [13,14]). Changing the expression of any one of these genes, like for example *MIG2*, may explain the *rnt1∆* phenotypes, like the perturbation of mitochondrial functions [57], the induction of galactose controlled genes [57] and impaired aerobic metabolism [58]. Since Rnt1p is not essential for growth in either aerobic or anaerobic condition, we conclude that the role of Rnt1p is most likely to fine-tune the expression of the genes involved in the respiro-fermentative flux.

## Methods

### Yeast culture and total RNA extraction

The wild type haploid strain *RNT1* (*MATa*, *lys2∆0*, *ura3∆0*, *his3∆200*, *leu2∆0)* and the haploid *rnt1∆* strain (*MATa*, *lys2∆0*, *ura3∆0*, *his3∆200*, *leu2∆0*, *rnt1*::*HIS3*) were generated by the replacement of one *RNT1* allele by *HIS3* in the *LLY36* diploid strain (*MATa/α lys2∆0/lys2∆0 ura3∆0/ura3∆0 his3∆200/his3∆200 leu2∆0/ leu2∆0*), followed by spore dissection as previously described [26]. The starting *LLY36* diploid strain was obtained by mating the strains *BY4700* (*MATa*, *ura3∆0*) and *BY4705* (*MATα*, *ura3∆0*, *leu2∆0*, *lys2∆0*, *ade2∆*::*hisG*, *his3∆200*, *trp1∆63*, *met15∆0*) [59], followed by spore dissection, as previously described [60]. The resulting haploid spores of each mating type and having the desired markers (*lys2∆0*, *ura3∆0*, *his3∆200*, *leu2∆0*) were selected and crossed together to yield the diploid homozygous *LLY36* strain. The *rrp6∆* strain (*MATa*, *his3∆1 leu2∆0*, *met15∆0*, *ura3∆0*, *rrp6∆*::*KMX4*) was taken from the Yeast knock out collection obtained from Open Biosystems [61]. Yeast cells were grown and manipulated using standard procedures [62] in YEP media supplemented with 2% dextrose at 26°C (the permissive temperature for *rnt1∆* strains). In **Fig. 7B**, cells were grown in YEP media supplemented with 2% dextrose or 4% galactose. In the case of the nitrogen shift experiments, assays were performed using cells grown in 600 ml of semisynthetic medium (SSD) containing per liter, 3 g of yeast extract, 10 g of dextrose, 0.8 g of NH_4_SO_4_, 1 g of KH_2_PO_4_, 0.5 g of NaCl, 0.5 g of CaCl_2_•2H_2_O, 0.3 g of MgSO_4_, 1.1 μg of FeCl_3_•6H_2_O, supplemented with amino acids, adenine and uracil at 40 μg/ml, 0.1% (V/V) Tween 80, 20 μg/ml of ergosterol and 350 ppm of antifoam B emulsion (Sigma-Aldrich, St. Louis, MO) [63] using Multifors (Infors Canada, Anjou, QC, Canada) bioreactors. Cells were grown to 0.3 OD_600_ in air-supplemented media than the gas supply was shifted to nitrogen. Forty ml samples were collected at different time points and the cells rapidly harvested by filtration. In **Fig. 7C-E**, strains were transformed either with an empty plasmid (pRS316) or expressing the wildtype *RNT1* allele (pRNT1) and grown in YC-ura media. Growth rates of *RNT1* and *rnt1∆* cells grown in presence of different carbon sources was performed and calculated as described [60].

### *In vitro* cleavage of total RNA

The cleavage assays were performed as previously described [36] with few modifications. Briefly, 30 μg of total *rnt1*∆ RNA was incubated with 6 pmol of purified Rnt1p for 20 min at 30°C in 100 μl of reaction buffer [30mM Tris–HCl (pH 7.5), 5mM spermidine, 0.1mM DTT, 0.1mM EDTA (pH7.5), 10mM MgCl_2_, 150mM KCl]. The reactions were stopped by phenol-chloroform extraction, and the RNA recuperated using salted ethanol precipitation.

### Synthesis and cleavage of small RNA hairpins

RNA substrates were synthesized using T7 RNA polymerase, radiolabeled and cleaved as described [22]. Three GC base pairs were added at the end of each stem to improve transcription efficiency and structure stability. Briefly, trace amount of radiolabelled substrates (150 cpm/μl) was incubated with 30 nM Rnt1p for 10 min at 30°C in 20 μl reaction buffer [30 mM Tris-HCl (pH 7.5), 5 mM spermidine, 0.1 mM DTT, 0.1 mM EDTA, 10 mM MgCl_2_ and 10 mM KCl]. Cleavage products were separated on 20% denaturing PAGE and visualized using a Storm 860 imager (GE Healthcare).

### RNA detection and analysis

Northern blots were performed as described [27] using 15 μg of total RNA and a 1% denaturing agarose gel. The RNA was visualized by autoradiography using randomly labeled probes corresponding to each of the genes examined. 5’-end-labeled oligonucleotide probes were used for detecting snR85 and 5S rRNA. The radiolabeled bands were visualized using a Storm 860 scanner (GE Healthcare) and analyzed using the ImageQuant software (Molecular Dynamics). The primer extension reactions were performed as described [36] using gene specific primers. cDNA synthesis and real-time PCR quantification of relative mRNA expression was performed as described [60] using a Biorad CFX384 Real-Time PCR Detection System. The Ct values of each gene were normalized to the values obtained for the ACT1 mRNA in each samples. The change in gene expression was calculated relative to the values obtained for wild-type RNA. All experiments were performed using at least three independent cultures and the PCR reactions conducted in duplicates. Genes with over 1.2 fold change and p < 0.01 (one-tailed t test) were considered as statistically overexpressed. The list of oligonucleotides used to generate Northern blot and primer extension probes, as well as qPCR reactions, can be provided upon request.

### *In silico* prediction of Rnt1p cleavage motifs

The method used for the prediction of Rnt1p cleavage signals is adapted from an earlier version used for the prediction of snoRNA cleavage signals [25]. The modified algorithm assigned positional weights based on the level of nucleotides conservation in closely related *Saccharomyces* species (sensu stricto). The outline of the method is shown in **S1A Fig.** Nucleotide conservation (**Fig. 6**) was calculated using Fisher test, while base pairing conservation was calculated using chi-squared test p value < 0.05 and both values were adjusted using Bonferroni correction.

### Expression array analysis and data treatment

The cDNA preparations and biotin end labeling were performed using total RNA by the University of Wisconsin Gene Expression Center (http://www.biotech.wisc.edu/services/gec). Array hybridization was performed at the Centre for Applied Genomics at University of Toronto (http://www.tcag.ca/index.html) according to the protocols supplied with Affymetrix GeneChip WT Double-Stranded Target Assay (without amplification) and Affymetrix GeneChip *S*. *cerevisiae* Tiling 1.0R Array (Affymetrix; PN: 900645). Probes were annotated using the *S*. *cerevisiae* S288c reference genome (SGD, http://www.yeastgenome.org, August 10th, 2007) as described [16]. The array was performed only once for each condition tested. The raw microarray data was treated as described [16] for reference DNA correction, variance stabilization and normalization using the tiling Array R package and in-house scripts. In addition, variations in probe intensity were corrected based on the predicted hybridization ∆G (**S2B Fig.**). The signals from the top 5% of the probes with the highest hybridization ∆G were removed and the signal intensities of the remaining probes were adjusted to obtain a null slope and a null average variation. The variation in intensity between *RNT1* and *rnt1∆* samples was analyzed using Huber segmentation algorithm [64] with consideration of the BIC (Bayesian Information Criterion) optimal number of segment. The level of variation in expression was defined as the median level of variation in all probes within the region. Neighboring segments with more than 2 fold overexpression and less than 48 nucleotides apart were joined together to form a single segment. Segments with less than 12 uniquely matching probes were considered unreliable and removed from further analyses. Overlapping features (e.g. gene name) were identified using SGD reference genome (http://www.yeastgenome.org, August 10th, 2007). Expression values were adjusted so that the deleted auxotrophic genes display the lowest expression level. Comparison between the array and quantitative RT-PCR (**S2C Fig.**) resulted in Spearman correlation coefficient of 0.798 (p < 2.2E-16, n = 202).

Different methods of data normalization were examined to identify the best strategy for reducing the potential effects of changes in the expression of abundant RNAs like non-coding RNA. The microarray data were normalized using different methods: 1) relative to known unaffected genes (e.g. the constitutively expressed RNAPII transcribed gene, ACT1, and the RNAPIII transcribed genes, U6, RPR1, RNA170 and SCR1), 2) relative to the expression level of intergenic regions, 3) relative to both unaffected genes and intergenic regions or 4) using the most robust parameter (lts.quantile = 0.5) of the classical VSN algorithm [64] (S6 Table). As shown, in new S3 Fig., the different normalization methods produced similar expression graphs and exhibited similar correlation with the data obtained using quantitative RT-PCR (S3E Fig.). However, the array data normalization used in Fig. 2 more accurately represented the values obtained by quantitative RT-PCR. Indeed, the correlation between this array and the quantitative RT-PCR were much better than previously published Rnt1p dependent conventional expression array [25,65]. These earlier arrays displayed a Spearman correlation coefficients with the quantitative RT-PCR data of 0.4 and 0.544 with, respectively. Similarly, the Spearman correlation factors resulting from comparing these earlier arrays to the tiling array performed in this study was 0.44 and 0.41 reflecting the low PCR reproducibility of the earlier array and differences in strains and growth conditions. The genes displaying similar Rnt1p dependent changes in the different array are indicated in **S6 Table** and **S7 Table**.

### Identification of Rnt1p cleavage events using tiling arrays (Cut and Chip)

Fifty μg of total RNA cleaved with recombinant Rnt1p was incubated with 8 μl Terminator 5´-Phosphate-Dependent Exoribonuclease (Xrn1p; Epicentre Biotechnologies, Madison, WI) for 90 min in the supplied buffer. The reactions were stopped by phenol-chloroform extraction, and the RNA collected using salted ethanol precipitation. Preparation of the cDNA, biotin labeling and chip hybridization was performed at the Centre for Applied Genomics at University of Toronto (http://www.tcag.ca/index.html) as described above. Microarray data was analyzed as described for expression array using the variation between treated and untreated samples for segmentation. Segments with less than 12 uniquely matching probes were removed. The median and the MAD (median absolute deviation) of the remaining segments were used to choose an appropriate cutoff (median less 1.96 times the MAD = -0.2425). Neighboring segments with a level below the chosen cutoff were grouped if separated by less than 48 nucleotides and regions smaller than 125 nucleotides were removed. The resulting 237 genomic regions were assigned to annotated features in the SGD genome of August 10th, 2007 (http://www.yeastgenome.org).

### Isolation and parallel sequencing of Rnt1p cleavage products (SALI)

Hundred μg of RNA cleaved with Rnt1p were purified using the mirVana miRNA Isolation Kit (Ambion, Life Technologies, Burlington, ON) to isolate RNA shorter than ~150 nucleotides. The enrichment of short RNAs was confirmed using the Agilent 2100 Bioanalyzer Small RNA kit (Agilent technologies, Santa Clara, CA). The cDNA libraries were generated from 500 ng of size selected RNA using the Ion Total RNA-Seq Kit v2. The IonTorrent sequencing data were generated using Ion 318 Chip Kit and acquired using Ion PGM System and Torrent Suite 2.2 software. 5' adapter sequences were trimmed using cutadapt 1.2rc2 [66]. Sequences shorter than 16 nucleotides were removed and the remaining reads aligned to *S*. *cerevisiae* reference genome sequence R64-1-1 using subread 1.3.5p4 [67]. Sequences with multiple matching positions were removed and reads ranging between 32 and 38 nucleotides were considered for further analysis. Read clusters consisting of 14 or more identical reads found in the cleaved and not the control samples were considered enriched. Enriched clusters of identical reads with over 50% overlap were merged and the resulting clusters were assigned to the transcripts with corresponding sequence. The RNA secondary structure for the longest merged cluster was predicted using Vienna RNA tools version 1.8.5. Wobble base pairs and non-canonical A-C base pairs were permitted in the predicted structures.

### Detection of Rnt1p cleavage in whole cell extracts

Exponentially growing *rnt1∆* cells were harvested and washed twice in AGK buffer (10 mM HEPES pH 8.0, 1.5 mM MgCl_2_, 200 mM KCl, 10% Glycerol) containing protease inhibitors. Cell pellet was resuspended in 1 volume of the same buffer and the slurry was quickly frozen in liquid nitrogen. About 12 grams of the frozen cell suspension was lysed in a 6870 Freezer Mill (SPEX SamplePrep). Grinded powder was then thawed on ice and spun at 20 000g for 30 minutes. The supernatant (S20 fraction) was collected and stored in aliquots at -80°C. Cleavage reactions were performed as described above except that total RNA was replaced by S20 extract (the amount was determined based on the measured RNA content in the extract). Detection of the cleavage products was performed as described for the Cut and Chip method.

### Detection of Rnt1p cleavage product *in vivo*

The terminal 5’-phosphates of the 3’ cleavage products identified by SALI and those near the stem-loops predicted by Cut and Chip were compared to those detected in the *xrn1∆ / dcp2∆* cells using global 5’ RACE (5’ RACE data was obtained from [35]). The RACE detected 5’-phosphates found within the first 5 nucleotides of the 3’ end of the cleavage products were considered a match and used for the generation of **Fig. 5E**.

### Cytometric assessment of respiratory competency

Yeast mitochondrial membrane potential was evaluated as previously described with few modifications [68]. Briefly, 4 x 10^6^ cells obtained from an exponentially growing culture were harvested and washed 3 times in PBS solution. Cells were stained with 35 μg / ml Rhodamine 123 for 10 minutes at 26°C or with 500 nM MitoTracker green FM for 50 minutes at the same temperature. Stained cells were washed 2 times with PBS and incubated for 15 minutes at 26°C in PBS. The resulting cells were further stained with 0.1 μg / ml propidium iodide in PBS and analyzed using a Fortessa cytometer (BD Biosciences, Mississauga, ON, Canada) equipped with a 50 mW solid state 488 nm laser. The emitted fluorescence of the Rhodamine123 and the MitoTracker green FM were detected at 530 ± 15 nm, while the propidium iodide detected at 610 ± 10 nm. Forward and side scatter signals were used to exclude debris and cell clumps. Dead cells were identified with propidium iodide staining and excluded from the analysis. For each sample, a minimum of 8 000 positive events by sample were acquired. Fluorescence intensity distribution profiles were traced using Cyflogic software (CyFlo Ltd, Finland) and raw data were exported and an analyzed as previously described [40].

### Microscopy

Cells grown to a density of 10^7^ cells / ml in YEPD were stained with 500 nM MitoTracker green FM for 30 minutes at 26°C in growth medium. Stained cells were washed two times in PBS and the mitochondria were visualized using 100 X / 1.46 oil objective with an excitation filter of 470 ± 20 nm and an emission filter of 540 ± 25 nm attached to the Zeiss Axio Observer microscope. Stacks were acquired at 200 nm intervals and deconvoluted using Zeiss Zen iterative algorithm. Maximum intensity projections of the deconvoluted images are shown.

### Measurements of NADH oscillations

NADH oscillations were measured as previously described [41]. Briefly, 100 ml of YC media buffered at pH 5 with 100 mM potassium phthalate and supplemented with 1% dextrose were inoculated to an OD_600_ of 0.2 using fresh saturated pre-cultures grown in the same media. Wild type and *rnt1∆* cells were grown 16–18 or 36–40 hours, respectively, at 26°C to deplete the sugar in the media. The resulting cells were washed twice with 10 ml of 50 mM potassium phosphate buffer pH 6.8 and finally suspended to 10% wet weight in the same buffer. Suspended cells were incubated 3 hours at 26°C before measuring the NAD / NADH fluorescence in a PTI spectrofluorometer using 3 ml of cell suspension at 30°C in a 4.5 ml PMMA cuvette. Cells were agitated for 5 minutes in the spectrofluorometer before data acquisition. Two readings per seconds were acquired with excitation at 366 nm and emission at 450 nm during 2 minutes for baseline recording before inducing oscillations with 30 mM glucose and 5 mM KCN.

### Calculation of gene ontologies enrichment

Enriched gene ontologies were detected by standard hypergeometric tests using the GOstats R package (version 2.18.0) [69] and annotation packages version 2.5.0. A Bonferroni corrected p-value of 0.05 was used to select significantly enriched terms. Background gene set contained the top 95% highly expressed mRNAs in *rnt1∆* strain.

### Accession numbers and raw data access

Raw and processed data presented in this study was deposited in the Gene Expression Omnibus (GEO, http://www.ncbi.nlm.nih.gov/geo/), under accession number GSE57450.

## Supporting Information

S1 Fig (related to Fig. 1) *In silico* prediction of Rnt1p cleavage signals. (**A**) Pipeline for the identification of Rnt1p substrates *in silico*. The relative weight of each parameter used for determining the final score is shown between parentheses. (**B**) *In silico* algorithm identifies Rnt1p reactive stem-loop structures. 24 stem-loops identified *in silico* spanning scores between 0.85 and 1.00 were T7-transcribed and tested for Rnt1p cleavage *in vitro* (see also **S3 Table**). The pie chart shows the proportion of targets for which Rnt1p cleavage was observed. (**C**) RNA degradation is not limited by the evolution of Rnt1p cleavage signals. Rnt1p cleavage signals are not restricted to genomes expressing the enzyme (*S*. *cerevisiae*) but extend to genomes expressing enzymes with alternative substrate specificity (*S*. *pombe*). Strikingly, the cleavage signals are frequently found in random sequence where di-nucleotide frequency and GC content are comparable to S. cerevisiae genome. The table indicates the total number of signals detected in each genome as well as the average number of loops per kilobase. (**D**) RNA degradation is determined by the context surrounding the cleavage signal. The predicted stem-loop structure, which failed to induce the cleavage of *POM33* mRNA (**Fig. 1G**), was independently produced by T7 RNA polymerase and tested for cleavage by Rnt1p as described in **Fig. 4F**. The position of the substrate (S) and cleavage products (P) is indicated on the right. Size markers (M) are shown to the left of the gel. The predicted hairpin structure is shown on the left and the position of the detected cleavage is indicated by an arrow. The asterisk indicates cleavage at non-canonical sites.
(TIFF)

S2 Fig (related to Fig. 2) Detection and normalization of gene expression profiles. (**A**) Pipeline for the identification of RNA segments overexpressed in *rnt1∆* cells. Data were obtained from Affymetrix yeast genomic tiling arrays hybridized to cDNA generated from wild type and *rnt1∆* total RNA. (**B**) Examples of GC contents adjustments and signal normalization. Signals from the tiling array (i) were adjusted according to the GC content (ii) of each probe and normalized relative to the median signals (iii). The data shown represent the signals obtained from probes hybridizing to the 250 nucleotides before and after MIG2 cleavage site (*MIG2* SNR). (**C**) Validation of the microarray data using quantitative PCR. The expression of 202 genes representing different levels of expression upregulation in *rnt1∆* cells were examined using quantitative RT-PCR and presented in the form of a dot plot relative to the array predicted expression levels (see also **S6 Table**). The coefficient of determination (R^2^) and Spearman Rho correlation (ρ) values between the two methods are shown on top. (**D**) Rnt1p is required for the removal of snoRNAs external transcribed spacers (ETSs). The line graphs illustrate the log_2_ change in snR48, snR50 and snR69 ETS levels after *RNT1* deletion. The nucleotide positions are shown relative to the snoRNA mature 5’ end. A schematic of each gene is shown on top and the position of known Rnt1p cleavage site is indicated as hairpins.
(TIFF)

S3 Fig (related to Fig. 2) Comparison between the normalization methods for the expression data. (**A**) Data was normalized relative to the signal obtained for 5 genes (constitutively expressed gene *ACT1*, and Pol III transcribed genes *U6*, *RPR1*, *RNA170* and *SCR1*) which are expected not to be affected by *RNT1* deletion. (**B**) Data was normalized relative to the signal obtained for all intergenic regions, excluding the 5% most affected in absence of *RNT1*. (**C**) A combination of the methods used in B and C was used to normalize the data. (**D**) Data was normalized using the "Variance Stabilization and Normalization" algorithm as described in Huber et al., Bioinformatics, 2002. Parameters were set to assume that 50% (Its.quantile = 0.5) of the probes don't vary in expression between *RNT1* and *rnt1∆* samples. (**E**) The results obtained with each normalization method were compared to the values obtained by quantitative PCR (see **S6 Table**) and the rho and median difference between array and qPCR were calculated.
(TIFF)

S4 Fig (related to Fig. 3) Detection and validation of Rnt1p degradation targets. (**A**) Pipeline for the identification of RNA segments cleaved by Rnt1p *in vitro*. Data were obtained using Affymetrix yeast genomic tiling array (see Fig. 3A). (**B**) *rnt1∆* RNA incubated in the absence or presence of Rnt1p was separated on agarose gel either directly or after treatment with Xrn1p. The RNA fragments were visualized using probes against two known RNA substrates (MIG2 and GPI17). *ACT1* and 5S rRNA were included as negative controls. The uncapped 25S rRNA was used as indicator of Xrn1p activity. Schemes of the mature transcript cleavage products and probe positions (dashed line) are respectively shown on the right. (**C**) Cleavage segments were separated based on the presence or the absence of G2-loops near the cleavage site and the distribution shown in the form of a pie chart. (**D**) Examples of RNAs in which reactivity was rated below the Cut and Chip detection cut off (false negatives). Segments were considered to be cleaved by Rnt1p only if their expression profile decreased below -0.2425 or more in order to reduce the number of false positives (see Methods section). However, a few genes like *ATP16* and *PAM1* showed weak (below cutoff), but distinguishable cleavage patterns upon manual observation. The line graphs show the degradation profile generated from the tiling array data as described in **Fig. 3E**. (**E**) Additional examples of Cut and Chip predicted substrates validated by primer extension as described in **Fig. 3E**.
(TIFF)

S5 Fig (related to Fig. 4) Detection of Rnt1p cleavage sites using SALI. (**A**) Pipeline for the detection of internal cleavage segments. (**B**) Characteristics of the sequencing reads obtained before and after treatment with Rnt1p. (**C**) SALI accurately detects the position of Rnt1p cleavage site. The sequence obtained by SALI (in bold) of 5 well-established cleavage sites was determined and aligned relative to the above corresponding RNA sequence. Primary and secondary cleavage sites are indicated by large and small arrows, respectively. The tetraloop sequence is underlined. The number of reads corresponding to the sequence shown in bold is indicated on the right. (**D**) Examples of the read coverage of Rnt1p cleavage signals. The read distribution near Rnt1p cleavage signals is illustrated in the form of a line graph. The nucleotide numbers are indicated relative to the start codon. No corresponding reads were detected in the untreated samples. (**E**) Additional examples of cleavage sites featuring 4, 5 and 6 nucleotides loops were synthesized using T7 RNA polymerase and tested for cleavage as described in **Fig. 3F**. (**F**) The G2-loop of the established U5 substrate was replaced by the newly identified 5-nt loop sequence found in OSH6 mRNA and tested for cleavage as described in **Fig. 4F**. The position of the mutations is shown by open and shaded boxes. (**G**) Northern blot analysis of substrates identified in **Fig. 4F**. Cleavage reactions and Northern blot analysis was performed as described in **Fig. 1G**.
(TIFF)

S6 Fig (related to Fig. 7) Effect of changes in growth conditions on the expression of Rnt1p substrates. (**A**) Effect of carbon source on the expression of Rnt1p and its substrates. The expression levels were determined using quantitative RT-PCR on RNA extracted from *RNT1* (black) and *rnt1∆* (grey) cells grown in 4% galactose (Gal) or 2% dextrose (Dex). The data were normalized relative to expression levels of *RNT1* cells grown in Dex and shown in the form of a bar graph. (**B**) Kinetics of gene expression of Rnt1p substrates after oxygen depletion. The expression levels of the different mRNA were determined using quantitative RT-PCR after shift to growth under nitrogen (N_2_). The expression levels detected in *rnt1∆* cells are shown relative to the levels detected in *RNT1* strain before N_2_ shift (t = 0 min). (**C**) The growth rates (in hours) were determined for *RNT1* and *rnt1∆* cells grown in presence of different carbon sources. The values were calculated from three independent cultures. (**D**) The growth rates of *RNT1* (black) and *rnt1∆* (white) cells calculated above were plotted relative to the growth of respective strains in media containing dextrose as carbon source.
(TIFF)

S7 Fig (related to Figs. 1, 2, 3 and 4) Table comparing the advantages and disadvantages of each of the different methods used to determine Rnt1p cleavage targets. (TIFF)

S1 Table (related to Fig. 1) List of published Rnt1p substrates considered in this study. (XLSX)

S2 Table (related to Fig. 1) Predicted G2-loop cleavage motifs with score above 0.70. (XLSX)

S3 Table (related to Fig. 1) *In vitro* cleavage of T7-transcribed tetraloops. (XLSX)

S4 Table (related to Fig. 1) List of transcripts containing at least one predicted G2-loop with scores higher than 0.85. (XLSX)

S5 Table (related to Fig. 1) Top 30 G2-loops. (XLSX)

S6 Table (related to Fig. 2) Comparison between the tiling arrays and quantitative RT-PCR detected changes in gene expression upon *RNT1* deletion (*RNT1* / *rnt1∆* expression (log_2_)). (XLSX)

S7 Table (related to Fig. 2) SGD annotated genes overexpressed in *rnt1∆* strain. (XLSX)

S8 Table (related to Fig. 2) SGD annotated genes overexpressed in *rrp6∆* strain. (XLSX)

S9 Table (related to Fig. 2) RNA segments overexpressed in *rnt1∆* strain. (XLSX)

S10 Table (related to Fig. 2) Top 30 overexpressed regions in *rnt1∆* strain. (XLSX)

S11 Table (related to Fig. 3) RNA degradation targets predicted by “Cut and Chip”. (XLSX)

S12 Table (related to Fig. 3) Top 30 cleavage targets predicted by “Cut and Chip”. (XLSX)

S13 Table (related to Fig. 4) Sequence reads enriched in Rnt1p treated RNA. (XLSX)

S14 Table (related to Figs. 2, 3 and 4) Estimation of false positive rate using 30 PCGs showing no cleavage by Northern blot. (XLSX)

S15 Table (related to Fig. 4) Cleavage targets predicted by SALI. (XLSX)

S16 Table (related to Fig. 4) Top 30 enriched read clusters predicted by SALI. (XLSX)

S17 Table (related to Fig. 5) Quantitative RT-PCR measurements of Rnt1p target's expression in different growth conditions (Relative expression upon *RNT1* deletion (log_2_)). (XLSX)

S18 Table (related to Figs. 2, 3 and 4) GO slim terms associated with Rnt1p dependent genes. (XLSX)

S19 Table (related to Figs. 2, 3 and 4) Gene ontology terms associated with Rnt1p dependent genes. (XLSX)

S20 Table (related to Figs. 1 to 4) Summary of the newly identified Rnt1p targets. (XLSX)
