# Supplementary figures and images for: Transcriptome Wide Annotation of Eukaryotic RNase III Reactivity and Degradation Signals
Source: PLoS Genet. 2015 Feb 13;11(2):e1005000. doi: 10.1371/journal.pgen.1005000 (PMC4334505; doi:10.1371/journal.pgen.1005000)

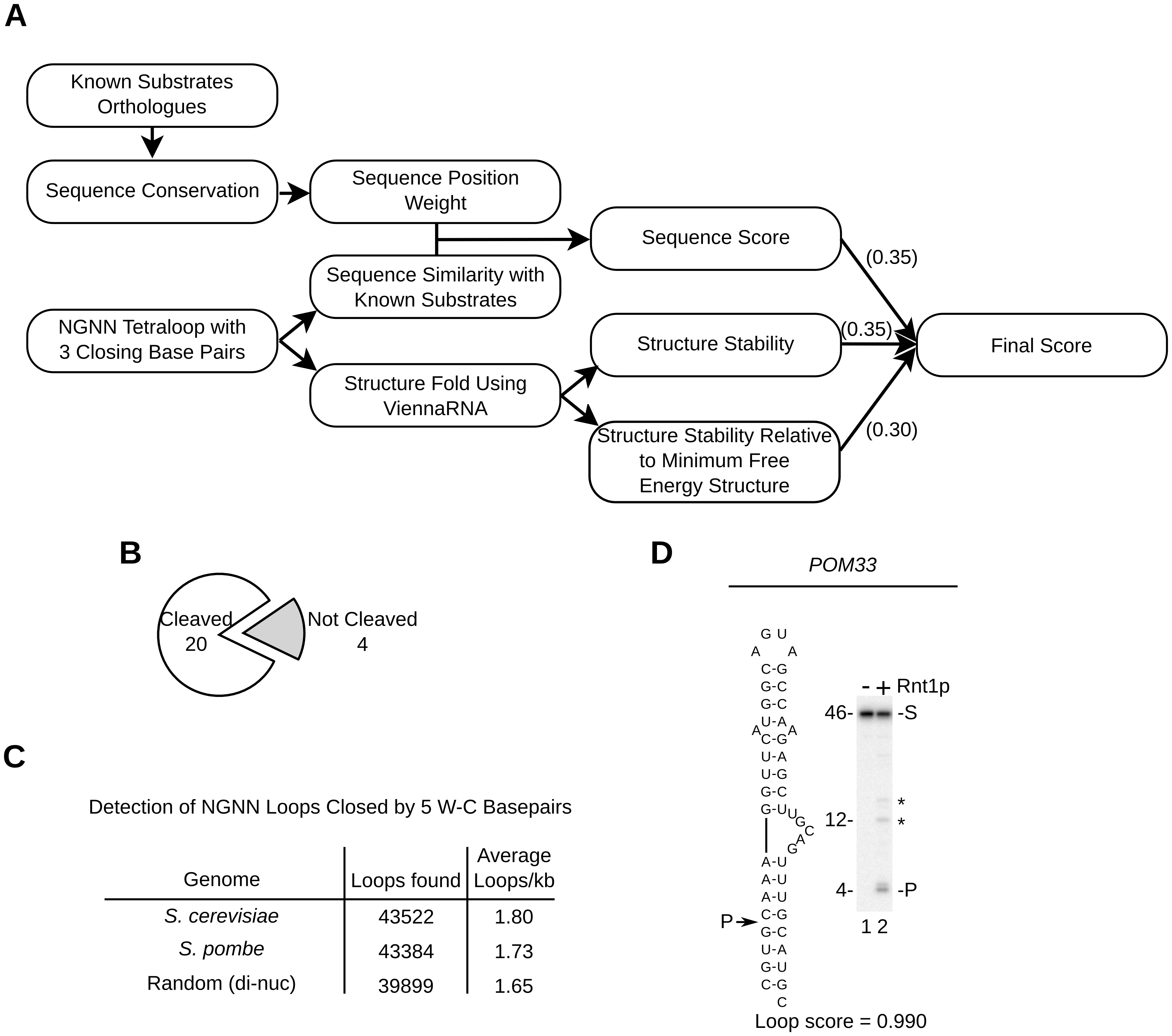

Supplement: S1 Fig — (A) Pipeline for the identification of Rnt1p substrates in silico. The relative weight of each parameter used for determining the final score is shown between parentheses. (B) In silico algorithm identifies Rnt1p reactive stem-loop structures. 24 stem-loops identified in silico spanning scores between 0.85 and 1.00 were T7-transcribed and tested for Rnt1p cleavage in vitro (see also S3 Table). The pie chart shows the proportion of targets for which Rnt1p cleavage was observed. (C) RNA degradation is not limited by the evolution of Rnt1p cleavage signals. Rnt1p cleavage signals are not restricted to genomes expressing the enzyme (S. cerevisiae) but extend to genomes expressing enzymes with alternative substrate specificity (S. pombe). Strikingly, the cleavage signals are frequently found in random sequence where di-nucleotide frequency and GC content are comparable to S. cerevisiae genome. The table indicates the total number of signals detected in each genome as well as the average number of loops per kilobase. (D) RNA degradation is determined by the context surrounding the cleavage signal. The predicted stem-loop structure, which failed to induce the cleavage of POM33 mRNA (Fig. 1G), was independently produced by T7 RNA polymerase and tested for cleavage by Rnt1p as described in Fig. 4F. The position of the substrate (S) and cleavage products (P) is indicated on the right. Size markers (M) are shown to the left of the gel. The predicted hairpin structure is shown on the left and the position of the detected cleavage is indicated by an arrow. The asterisk indicates cleavage at non-canonical sites. (TIFF) [file pgen.1005000.s001.tiff]

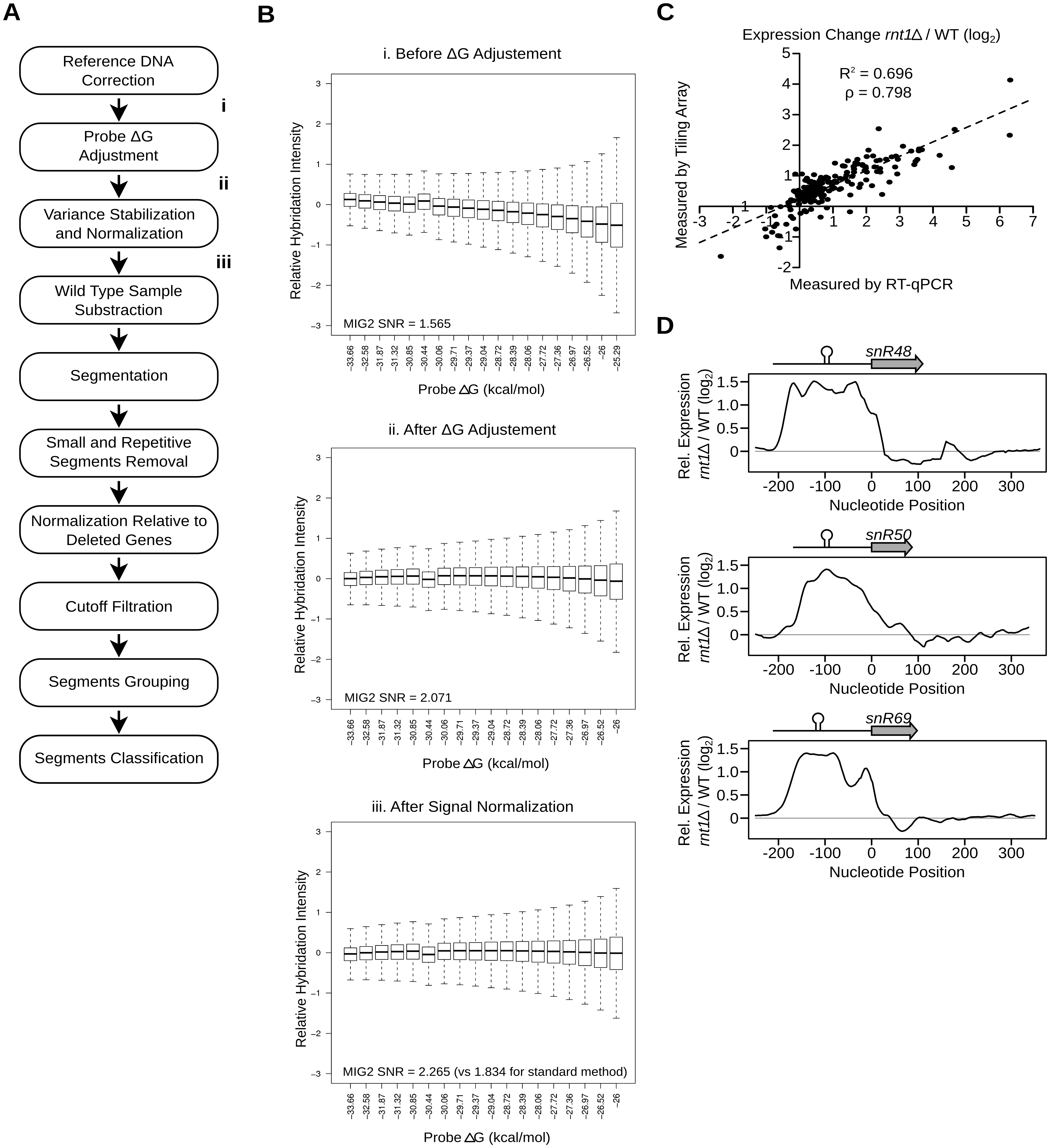

Supplement: S2 Fig — (A) Pipeline for the identification of RNA segments overexpressed in rnt1∆ cells. Data were obtained from Affymetrix yeast genomic tiling arrays hybridized to cDNA generated from wild type and rnt1∆ total RNA. (B) Examples of GC contents adjustments and signal normalization. Signals from the tiling array (i) were adjusted according to the GC content (ii) of each probe and normalized relative to the median signals (iii). The data shown represent the signals obtained from probes hybridizing to the 250 nucleotides before and after MIG2 cleavage site (MIG2 SNR). (C) Validation of the microarray data using quantitative PCR. The expression of 202 genes representing different levels of expression upregulation in rnt1∆ cells were examined using quantitative RT-PCR and presented in the form of a dot plot relative to the array predicted expression levels (see also S6 Table). The coefficient of determination (R2) and Spearman Rho correlation (ρ) values between the two methods are shown on top. (D) Rnt1p is required for the removal of snoRNAs external transcribed spacers (ETSs). The line graphs illustrate the log2 change in snR48, snR50 and snR69 ETS levels after RNT1 deletion. The nucleotide positions are shown relative to the snoRNA mature 5’ end. A schematic of each gene is shown on top and the position of known Rnt1p cleavage site is indicated as hairpins. (TIFF) [file pgen.1005000.s002.tiff]

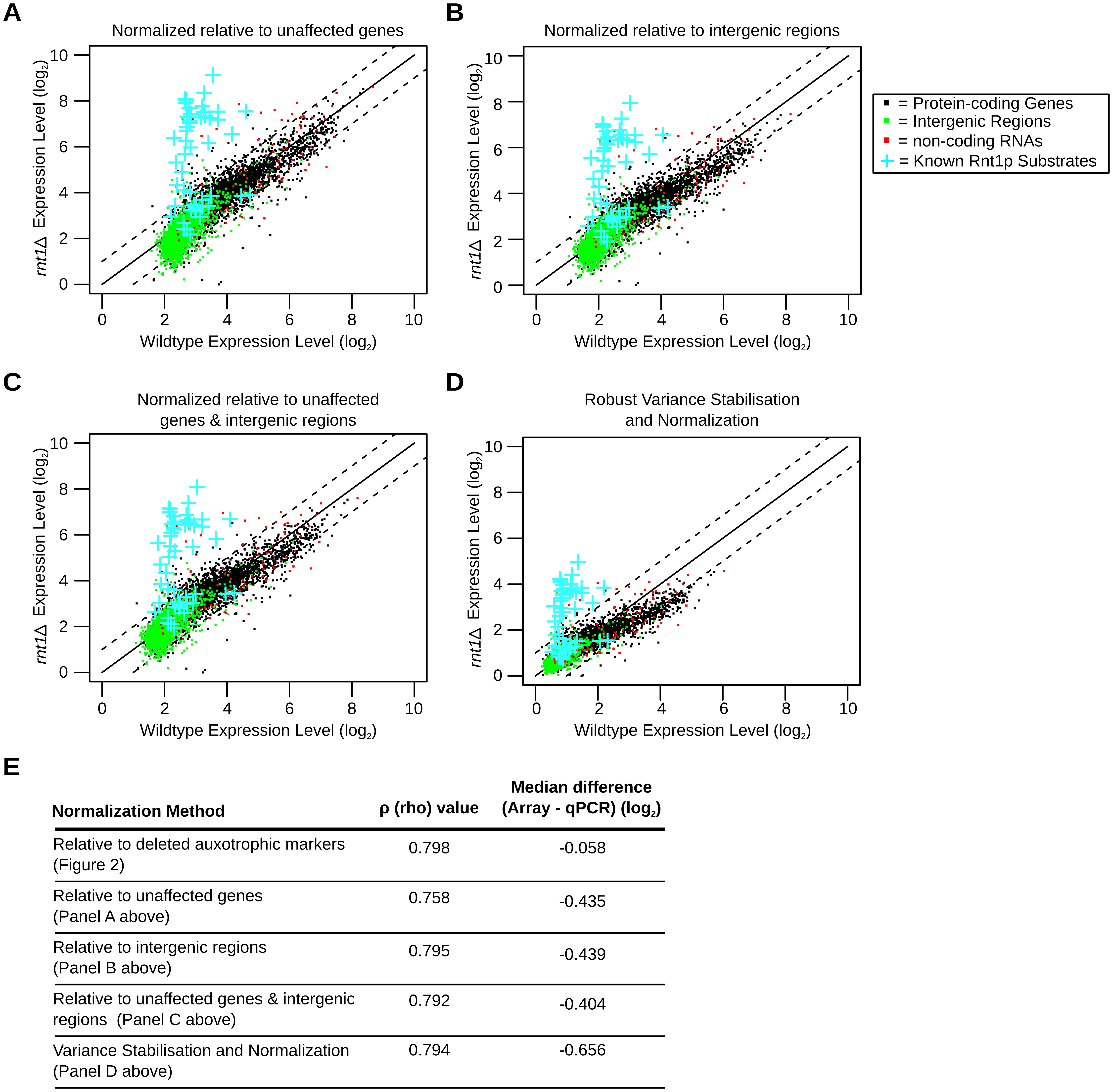

Supplement: S3 Fig — (A) Data was normalized relative to the signal obtained for 5 genes (constitutively expressed gene ACT1, and Pol III transcribed genes U6, RPR1, RNA170 and SCR1) which are expected not to be affected by RNT1 deletion. (B) Data was normalized relative to the signal obtained for all intergenic regions, excluding the 5% most affected in absence of RNT1. (C) A combination of the methods used in B and C was used to normalize the data. (D) Data was normalized using the "Variance Stabilization and Normalization" algorithm as described in Huber et al., Bioinformatics, 2002. Parameters were set to assume that 50% (Its.quantile = 0.5) of the probes don't vary in expression between RNT1 and rnt1∆ samples. (E) The results obtained with each normalization method were compared to the values obtained by quantitative PCR (see S6 Table) and the rho and median difference between array and qPCR were calculated. (TIFF) [file pgen.1005000.s003.tiff]

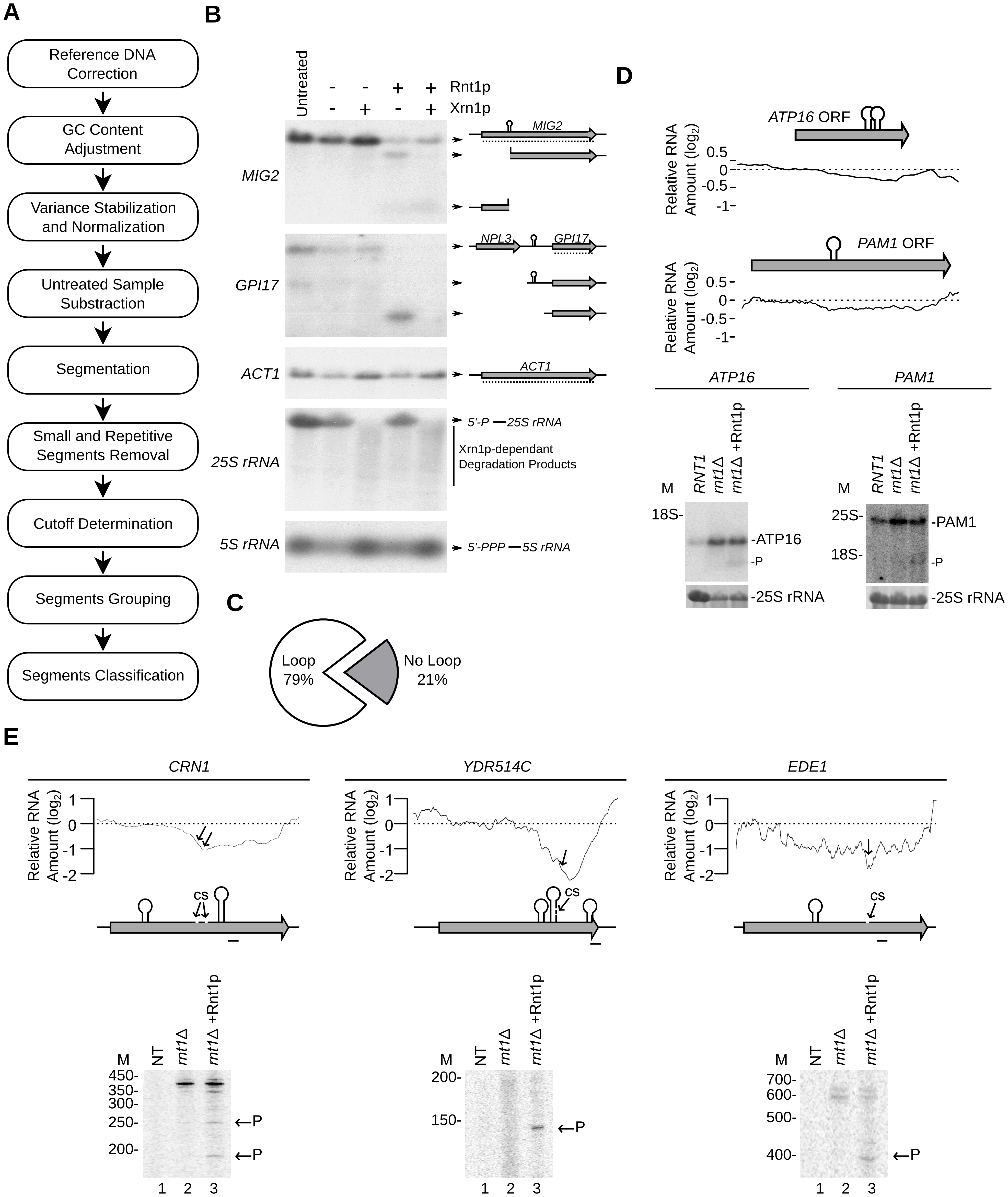

Supplement: S4 Fig — (A) Pipeline for the identification of RNA segments cleaved by Rnt1p in vitro. Data were obtained using Affymetrix yeast genomic tiling array (see Fig. 3A). (B) rnt1∆ RNA incubated in the absence or presence of Rnt1p was separated on agarose gel either directly or after treatment with Xrn1p. The RNA fragments were visualized using probes against two known RNA substrates (MIG2 and GPI17). ACT1 and 5S rRNA were included as negative controls. The uncapped 25S rRNA was used as indicator of Xrn1p activity. Schemes of the mature transcript cleavage products and probe positions (dashed line) are respectively shown on the right. (C) Cleavage segments were separated based on the presence or the absence of G2-loops near the cleavage site and the distribution shown in the form of a pie chart. (D) Examples of RNAs in which reactivity was rated below the Cut and Chip detection cut off (false negatives). Segments were considered to be cleaved by Rnt1p only if their expression profile decreased below -0.2425 or more in order to reduce the number of false positives (see Methods section). However, a few genes like ATP16 and PAM1 showed weak (below cutoff), but distinguishable cleavage patterns upon manual observation. The line graphs show the degradation profile generated from the tiling array data as described in Fig. 3E. (E) Additional examples of Cut and Chip predicted substrates validated by primer extension as described in Fig. 3E. (TIFF) [file pgen.1005000.s004.tiff]

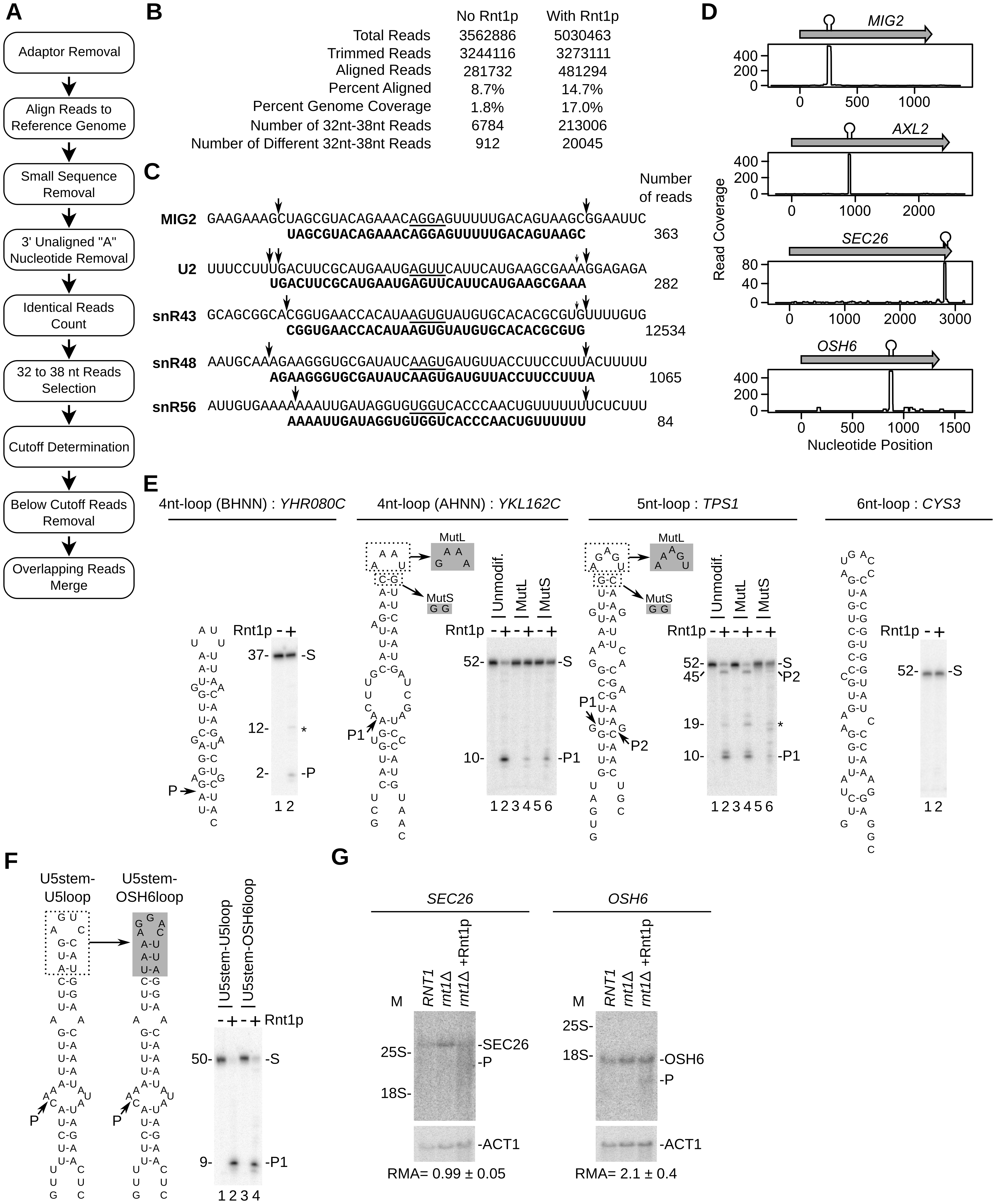

Supplement: S5 Fig — (A) Pipeline for the detection of internal cleavage segments. (B) Characteristics of the sequencing reads obtained before and after treatment with Rnt1p. (C) SALI accurately detects the position of Rnt1p cleavage site. The sequence obtained by SALI (in bold) of 5 well-established cleavage sites was determined and aligned relative to the above corresponding RNA sequence. Primary and secondary cleavage sites are indicated by large and small arrows, respectively. The tetraloop sequence is underlined. The number of reads corresponding to the sequence shown in bold is indicated on the right. (D) Examples of the read coverage of Rnt1p cleavage signals. The read distribution near Rnt1p cleavage signals is illustrated in the form of a line graph. The nucleotide numbers are indicated relative to the start codon. No corresponding reads were detected in the untreated samples. (E) Additional examples of cleavage sites featuring 4, 5 and 6 nucleotides loops were synthesized using T7 RNA polymerase and tested for cleavage as described in Fig. 3F. (F) The G2-loop of the established U5 substrate was replaced by the newly identified 5-nt loop sequence found in OSH6 mRNA and tested for cleavage as described in Fig. 4F. The position of the mutations is shown by open and shaded boxes. (G) Northern blot analysis of substrates identified in Fig. 4F. Cleavage reactions and Northern blot analysis was performed as described in Fig. 1G. (TIFF) [file pgen.1005000.s005.tiff]

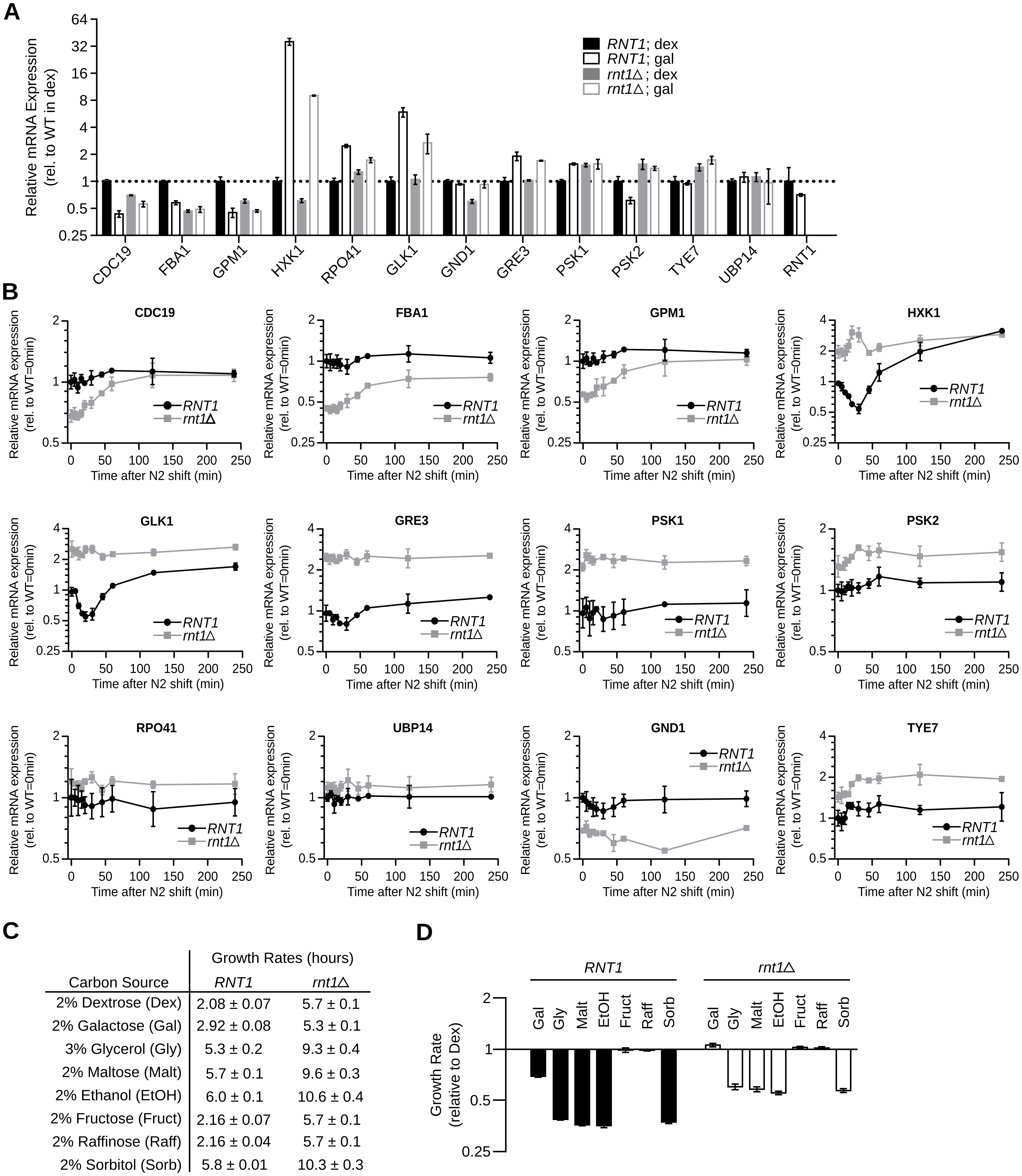

Supplement: S6 Fig — (A) Effect of carbon source on the expression of Rnt1p and its substrates. The expression levels were determined using quantitative RT-PCR on RNA extracted from RNT1 (black) and rnt1∆ (grey) cells grown in 4% galactose (Gal) or 2% dextrose (Dex). The data were normalized relative to expression levels of RNT1 cells grown in Dex and shown in the form of a bar graph. (B) Kinetics of gene expression of Rnt1p substrates after oxygen depletion. The expression levels of the different mRNA were determined using quantitative RT-PCR after shift to growth under nitrogen (N2). The expression levels detected in rnt1∆ cells are shown relative to the levels detected in RNT1 strain before N2 shift (t = 0 min). (C) The growth rates (in hours) were determined for RNT1 and rnt1∆ cells grown in presence of different carbon sources. The values were calculated from three independent cultures. (D) The growth rates of RNT1 (black) and rnt1∆ (white) cells calculated above were plotted relative to the growth of respective strains in media containing dextrose as carbon source. (TIFF) [file pgen.1005000.s006.tiff]

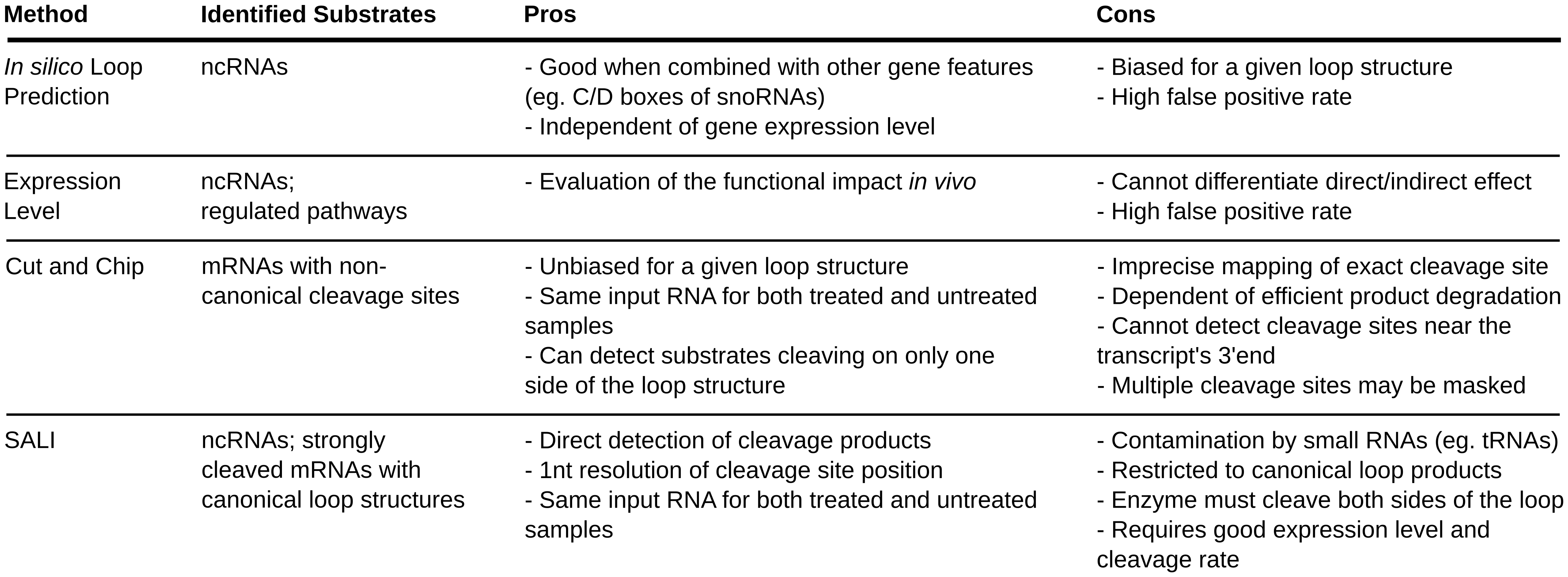

Supplement: S7 Fig — (TIFF) [file pgen.1005000.s007.tiff]
